# Thyroid hormone receptor beta mutations alter photoreceptor development and function in *Danio rerio* (zebrafish)

**DOI:** 10.1371/journal.pgen.1008869

**Published:** 2020-06-22

**Authors:** Ciana Deveau, Xiaodong Jiao, Sachihiro C. Suzuki, Asha Krishnakumar, Takeshi Yoshimatsu, J Fielding Hejtmancik, Ralph F. Nelson

**Affiliations:** 1 National Institute of Neurological Disorders and Stroke, National Institutes of Health, Rockville, Maryland, United States of America; 2 National Eye Institute, National Institutes of Health, Rockville, Maryland, United States of America; 3 Okinawa Institute of Science and Technology Graduate University, Okinawa, Japan; 4 University of Sussex, Brighton, United Kingdom; University of Idaho, UNITED STATES

## Abstract

We investigate mutations in *trβ2*, a splice variant of *thrb*, identifying changes in function, structure, and behavior in larval and adult zebrafish retinas. Two N-terminus CRISPR mutants were identified. The first is a *6BP+1* insertion deletion frameshift resulting in a truncated protein. The second is a *3BP* in frame deletion with intact binding domains. ERG recordings of isolated cone signals showed that the *6BP+1* mutants did not respond to red wavelengths of light while the *3BP* mutants did respond. *6BP+1* mutants lacked optomotor and optokinetic responses to red/black and green/black contrasts. Both larval and adult *6BP+1* mutants exhibit a loss of red-cone contribution to the ERG and an increase in UV-cone contribution. Transgenic reporters show loss of cone *trβ2* activation in the *6BP+1* mutant but increase in the density of cones with active blue, green, and UV opsin genes. Antibody reactivity for red-cone LWS1 and LWS2 opsin was absent in the *6BP+1* mutant, as was reactivity for arrestin3a. Our results confirm a critical role for *trβ2* in long-wavelength cone development.

## Author summary

There are four cone photoreceptors responsible for color vision in zebrafish: red, green, blue, and UV. The thyroid hormone receptor *trβ2* is localized in the vertebrate retina. We know that it is necessary for the development of cones expressing long-wavelength-sensitive opsins (red cones), but here we investigate the functional alterations that accompany a loss of *trβ2*. Our work contributes to the ongoing investigations of retinal development and the involvement of thyroid hormone receptors. As suggested by previous morphological findings, fish became red colorblind when *trβ2* was knocked out, and the contributions of the other three cone types shifted. Our work highlights the plasticity of photoreceptor patterning as we see changes in opsin peaks and cone sensitivity, increases in contributions of UV cones, and an attempt at a mosaic pattern in the adult retina, all in the absence of *trβ2* and red cones. We now have an increased understanding of mechanisms underlying retinal development.

## Introduction

To achieve vertebrate color vision, cones with different wavelength sensitivities develop from retinal progenitor cells. Cone signals stimulated by their respective wavelengths of light merge post-synaptically to create the visual perception of color in the brain. Proper differentiation of these cones, and subsequent proper color perception, is reliant on specific transcription factors. The vertebrate family of long-wavelength-sensing (LWS) cones are linked to thyroid hormone receptors. Differentiation of LWS cones in rodents requires an isoform of thyroid hormone receptor beta (*thrb*) called *trβ2* [1], one of two thyroid hormone receptors in a family of nuclear receptors [2]. Human heterozygous *thrb* mutants manifest a metabolic syndrome, resistance to thyroid hormone (RTHβ). In RTHβ there are color vision deficits [3]. In one reported human *thrb* homozygous mutant, both LWS and middle-wavelength-sensitive (MWS) cone functions were severely depressed, while short-wavelength-sensitive (SWS) cone function was enhanced [4]. Thyroid receptors (TRs) are ligand-dependent transcription factors that contain an N-terminus, a DNA binding domain, and a ligand (T3) binding domain, conserved across multiple vertebrates including chicken, mouse, human, and zebrafish [5, 6, 7, 8]. *Thrb* is broken down into multiple isoforms through alternative splicing, which are active in the vertebrate retina, pituitary gland, and inner ear [9]. The *trβ2* isoform is localized in the retina [9, 10].

Zebrafish (*Danio rerio*) is an important color vision model. It has four cone types, LWS opsin expressing (red cones), RH-2 opsin expressing (green cones), SWS2 opsin expressing (blue cones), and SWS1 opsin expressing (UV cones) [11]. Zebrafish has two opsins derived from the LWS evolutionary tree by gene duplication (*LWS1* & *LWS2*), and one from the SWS or UV-opsin evolutionary tree (*SWS1*). Humans also express two homologous LWS opsins (*LWS* & *MWS*), and one SWS opsin (*SWS*). In both species the two LWS opsins are in a tandem gene array governed by a common promoter region [12]. Each cone type expresses only one class of opsin in zebrafish [11]. *Trβ2* was previously shown in zebrafish to be essential for the development of red cones and, in an overexpression transgenic (*crx*:*trβ2)*, sufficient for inducing overexpression of LWS (red opsin) cones and a decrease in SWS1 (UV opsin) cones [10]. Of the cone types, expression of *trβ2* is initiated in progenitor cells of exclusively L-opsin expressing cones [10]. Our aim is to investigate germline alterations of *trβ2* expression. Although a morpholino knockdown of *trβ2* in 5-day larvae leads to a decrease of red cones and increase of UV cones [13, 10], it is unknown how that disrupts color vision functionally in larvae, leaves adult mutants unexplored, and does not address the phenotype for heterozygous mutants. This paper is the first to relate retinal physiology with behavior and to study the spectral tuning of retinal cone signals with respect to *trβ2* mutations.

To answer those questions, we established two *trβ2* mutant lines using the CRISPR/Cas9 system. The first is a *c*.*184_188delTATGGinsGTTCCC* (*6BP+1*) frameshift indel and the second is a *c*.*184_186delTAT* (p.Tyr61del) (*3BP*) a single in-frame codon deletion, both located in the first exon of the *trβ2* isoform, which is unique to this isoform and thus would be expected to affect only retinal development. The *6BP+1* frameshift mutation creates an early stop codon, eliminating the DNA binding site and ligand binding site. The *3BP* mutation deletes Tyr61, an N-terminus amino acid that is highly conserved among vertebrates, but leaves the DNA binding site and ligand binding site intact. The *6BP+1* mutation resulted in the anticipated LWS-cone loss, but the *3BP* mutation revealed unexpected results. Here we characterize the physiological differences through spectral electroretinograms (ERGs) of the cone signal (PIII) in zebrafish and changes in color perception through optomotor and optokinetic behavioral testing. Molecular changes are identified through antibody staining, transgenic reporters, and qPCR for key cone markers to provide a broad picture of the functional, behavioral, and molecular alterations. We found that the changes in the molecular makeup of the cone layer were reflected in both the ERG responses and behavior, supplementing our knowledge on the role of *trβ2* in the retina.

## Results

### CRISPR mutations in the N-terminus of *trβ2*

**Fig 1:**
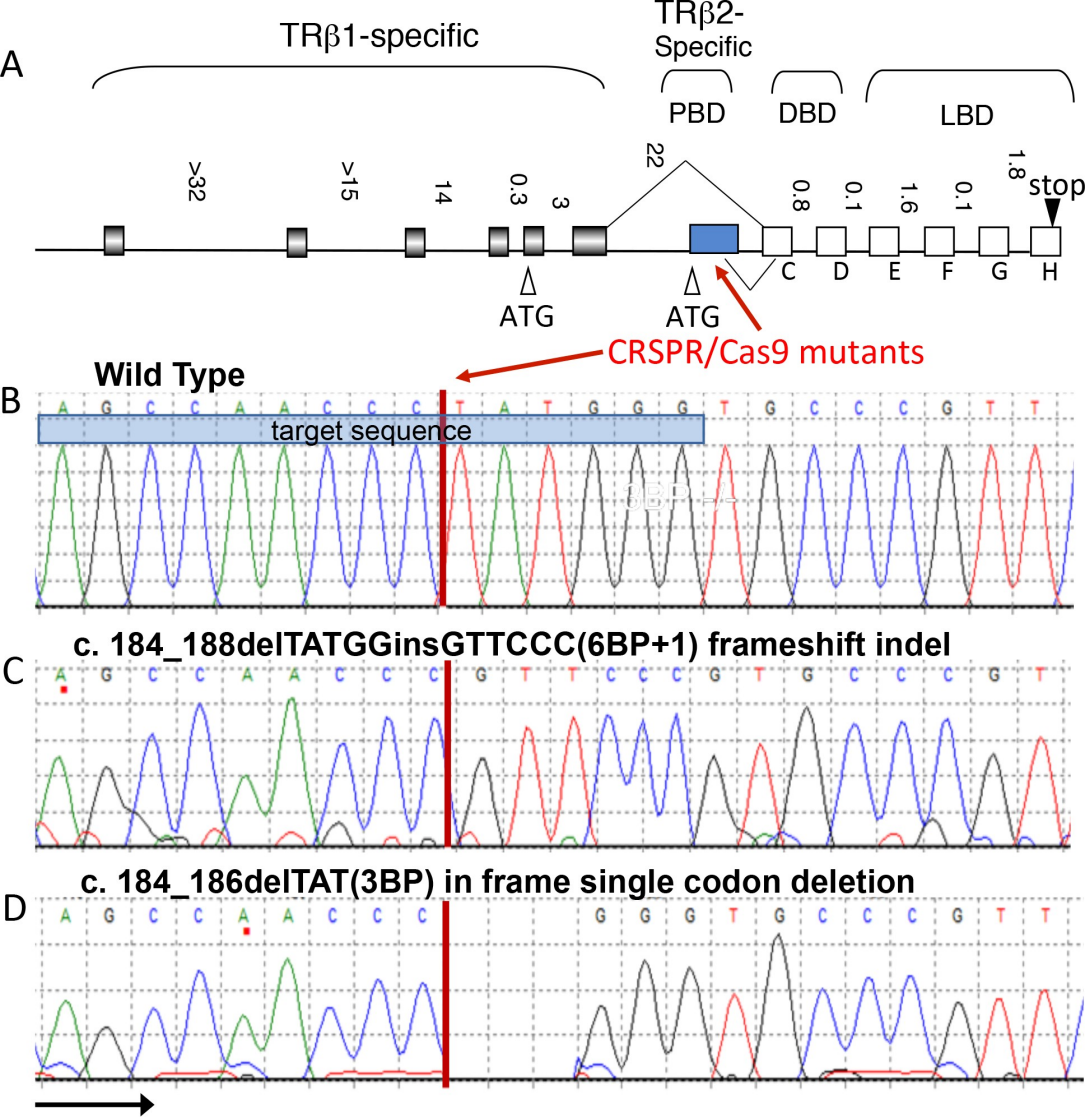
Two CRISPR mutant lines. (A) Both mutations are in the first exon of the *trβ2* isoform within the N-terminus in the protein binding domain (PBD). Modified from Jones et al. (2003) [14] with permission of *Thyroid*, Mary Ann Liebert, Inc., publishers, New Rochelle, NY. (B-D) Both mutations targeted the TAT codon resulting in a frameshift indel (*6BP+1*) and an in-frame codon deletion (*3BP*). DNA binding domain (DBD), ligand binding domain (LBD).

The two splice variants of the *thrb* gene, *trβ1* and *trβ2* differ only in the first exon (Fig 1A). To target *trβ2*, we designed gRNA targeting exon1 of *trβ2* and injected into one-cell-stage embryos of *Tg*(*trβ2*:tdTomato). We isolated two mutations of *trβ2*. Mutations were found at the CCC (proline) TAT (tyrosine) juncture. (Fig 1B–1D). The mutation in Fig 1C deletes a TATGG and replaces it with a GTTCCC, a frameshift indel mutation leading to a stop codon in the first exon. We refer to this as the *6BP+1* mutation. The mutation in Fig 1D deletes a single codon, TAT (tyrosine), from the *trβ2* sequence. We refer to this as the *3BP* mutation. The mutations occurred in the first exon of *trβ2*, in a region specifically involving the Tyr61 in the N-terminus, at the focus of the CRISPR/Cas9 targeting sequence. The *6BP+1* mutation eliminated both the DNA binding site (DBD) and thyroxin ligand binding site (LBD). The *3BP* mutation left both those regions intact but altered the protein binding site (PBD) at a highly conserved amino acid, possibly involved in transcription factor activation [5].

### Gene reporter activity for *trβ2* in *trβ2* mutants and heterozygous mutants

**Fig 2:**
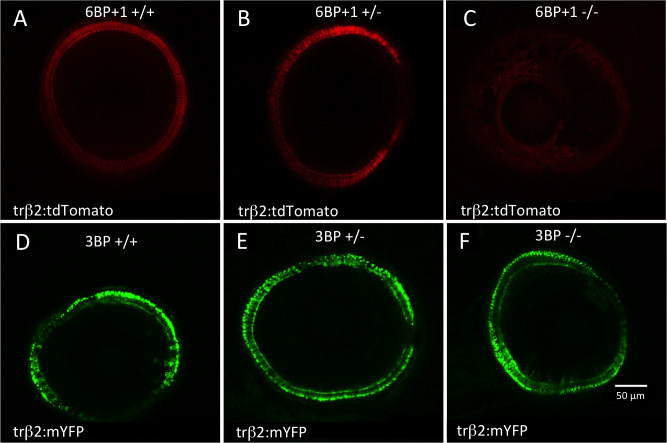
Live confocal imaging of larval eyes at 4- and 5-days post fertilization (dpf). (A) A confocal cross section of a wild type (*6BP+/+*) (N = 4) larval eye shows a ring of cone layer tdTomato fluorescence under control of a *trβ2* promoter in the red cones at the outer edge of the retina. (B) A *6BP+1*+/- heterozygote maintains (N = 7) *trβ2* fluorescence, but the *6BP+1-/-* mutant (N = 14) (C) has no *trβ2* fluorescence. The *3BP* wild type (N = 12) (D), heterozygote (N = 23) (E), and mutant (N = 5) (F) all exhibit *trβ2* fluorescence shown through mYFP expressed by a *trβ2* promoter. Eyes are imaged from the larval anterior side showing a cross-section ring of cones. Fluorescence in a ring or semi-ring formation indicates the presence of *trβ2* activity. Missing sections of the rings are due to image planes cutting through the cornea.

We hypothesized that the *trβ2* gene promotes its own transcription. Therefore, the absence of competent *trβ2* protein product was expected to diminish promoter activity for the *trβ2* gene. To test this hypothesis we crossed *6BP+1* homozygous and heterozygous fish carrying the reporter transgenes *trβ2*:*tdTomato* with *6BP+1* heterozygous fish. Similarly, we in-crossed *3BP* heterozygous fish carrying *trβ2*:*mYFP* [10]. Larvae were raised in PTU to block pigment epithelial melanin formation so that the fluorescent products of the reporter transgenes could be visualized within the larval eyes (4- and 5-dpf) using live confocal imaging (Fig 2). The transgenes produce fluorescent red-cone layers in the *6BP+1* WT (+/+, Fig 2A) and the *6BP+1* heterozygous mutant (+/-, Fig 2B) but not the *6BP+1* mutant (-/-, Fig 2C). In the *6BP+1* strain, all eyes fluorescent for tdTomato (N = 11) were either a heterozygous (+/-) mutant or wild type (+/+) genotype, while mutant (-/-) eyes failed to fluoresce (N = 14). This indicates that either a full (+/+) or half (+/-) dose of native *trβ2* is enough to activate the *trβ2* reporter gene, but the *6BP+1 trβ2* protein, truncated in the first exon, is not. Retinal diameters were measured as the maximal diameter of the cone fluorescent ring within the image stacks. In the non-fluorescent *6BP+1* mutant, diameter was measured from autofluorescence. There is no significant difference between the eye diameters of the *6BP+1* mutant (283.9 ±2.9 μm), heterozygote (283.6 ± 3.3 μm), and wild type (280.8 ± 2.1 μm) [ANOVA, F(2, 21) = 0.384, *P* = 0.686] or the *3BP* mutant (213.8 ± 9.5 μm), heterozygote (228.8 ± 5.1 μm) or WT (233.4 ± 2.9 μm) [ANOVA, F(2,18) = 2.94, *P* = 0.08].

In confocal imaging, *trβ2* reporter fluorescence occurred in all *3BP* mutants (N = 5) (-/-, Fig 2F), het mutants (N = 23) (+/-, Fig 2E), and wild types (N = 12) (+/+, Fig 2D). Fluorescent reporter activity based in the *trβ2* promoter is evident in all three genetic lineages of the *3BP* heterozygous in-cross. Despite the absence of the highly conserved tyrosine, the *3BP trβ2* protein activates the *trβ2* reporter.

### Immunohistochemistry on larval and adult retinas

**Fig 3:**
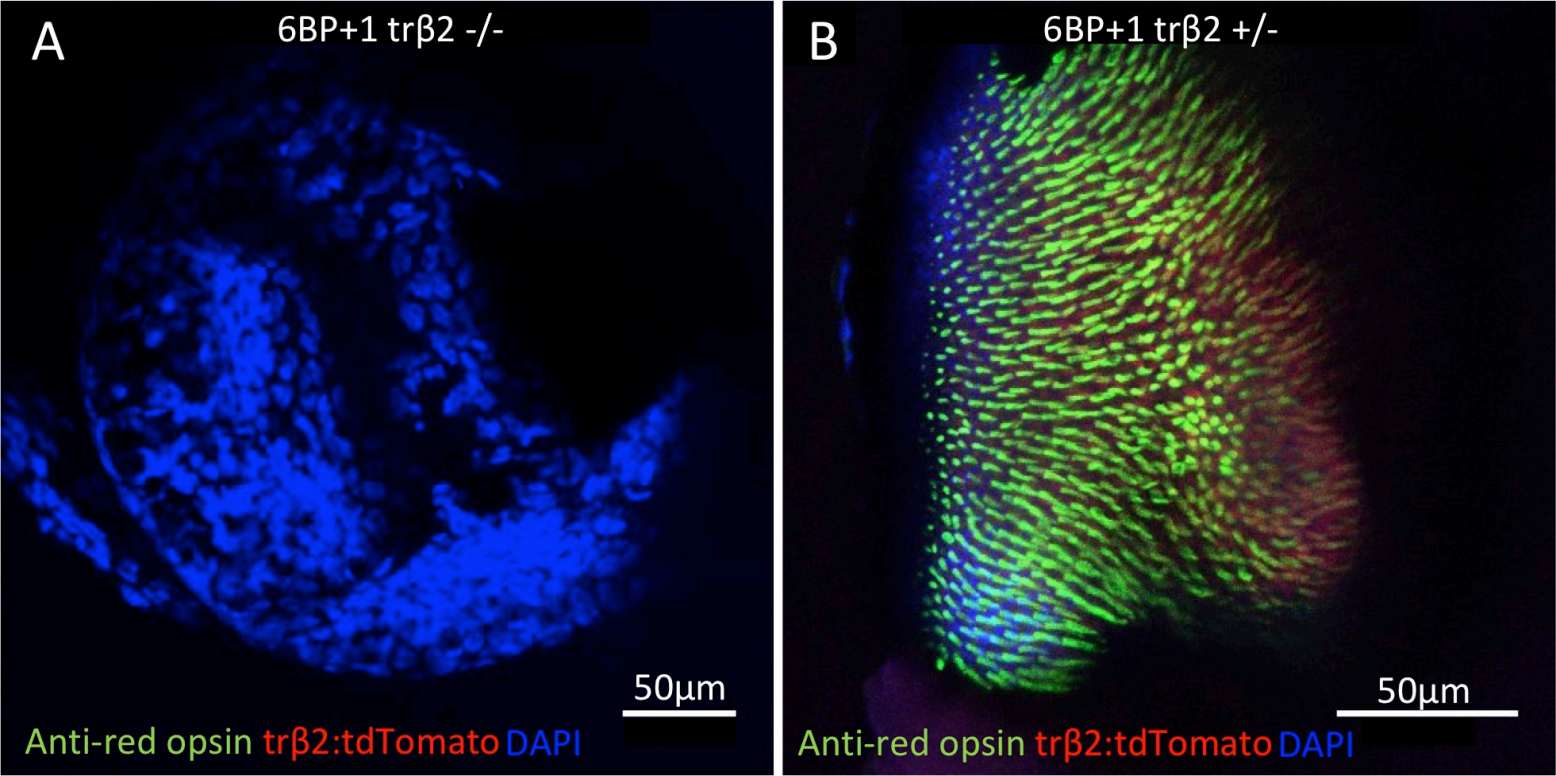
Antibody stains of 7 dpf larval retinas. (A) Mutant retina did not have anti-red-opsin or *trβ2* reporter fluorescence (N = 4). (B) Heterozygote retina showed the red opsin staining colocalized with tdTomato (N = 4). Images are taken from flattened retinas with the cone outer segments facing upward and in primary focus.

Larval retinas fixed at 7 dpf were stained for red opsin antigenicity on a DAPI background. The *6BP+1* mutant did not show red opsin antibody fluorescence or transgenic tdTomato fluorescence (Fig 3A). In contrast, the *6BP+1* heterozygote expressed red opsin antibody fluorescence in the outer segments of cells with *trβ2*:*tdTomato* cytoplasmic expression (Fig 3B). This 7 dpf mutant knockout result is consistent with previous morpholino knockdowns of *trβ2* in 5 dpf larvae [10, 13].

**Fig 4:**
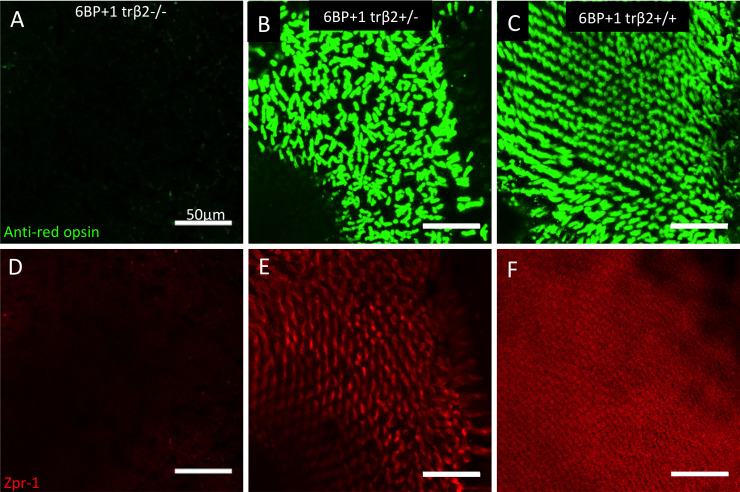
Anti-red opsin and zpr-1 antibody staining of *6BP+1* adult. (A, D) Mutant adult retinas (N = 4) did not have red opsin or arr3a (red-green double-cone) antibody fluorescence. (B, E) Heterozygote adult retinas (N = 4) showed bright antibody staining for red opsin and arrestin3a as seen in the wild type (N = 4) (C, F). Scale is the same for each image. Opsin and arr3a images are from the same microscope field, but different planes of focus. Staining was repeated twice for each genotype.

This loss of red-cone markers extends into adulthood. Fixed retinas of adult fish between 10- and 12-months post fertilization were stained with the arrestin (arr3a) antibody, zpr-1, known to mark both red principal and green accessory members of double cones and also a red-opsin antibody (1D4) marking only red cones [15, 16]. The *6BP+1* mutant had a complete loss of both cone markers (Fig 4A and 4D), while the heterozygote and wild type retinas have distinct arr3a and red-opsin fluorescence (Fig 4B, 4C, 4E and 4F). The heterozygote red-opsin cone staining is less uniform and the zpr-1 staining is less dense than the wild type (Fig 4B, 4C, 4E and 4F).

**Fig 5:**
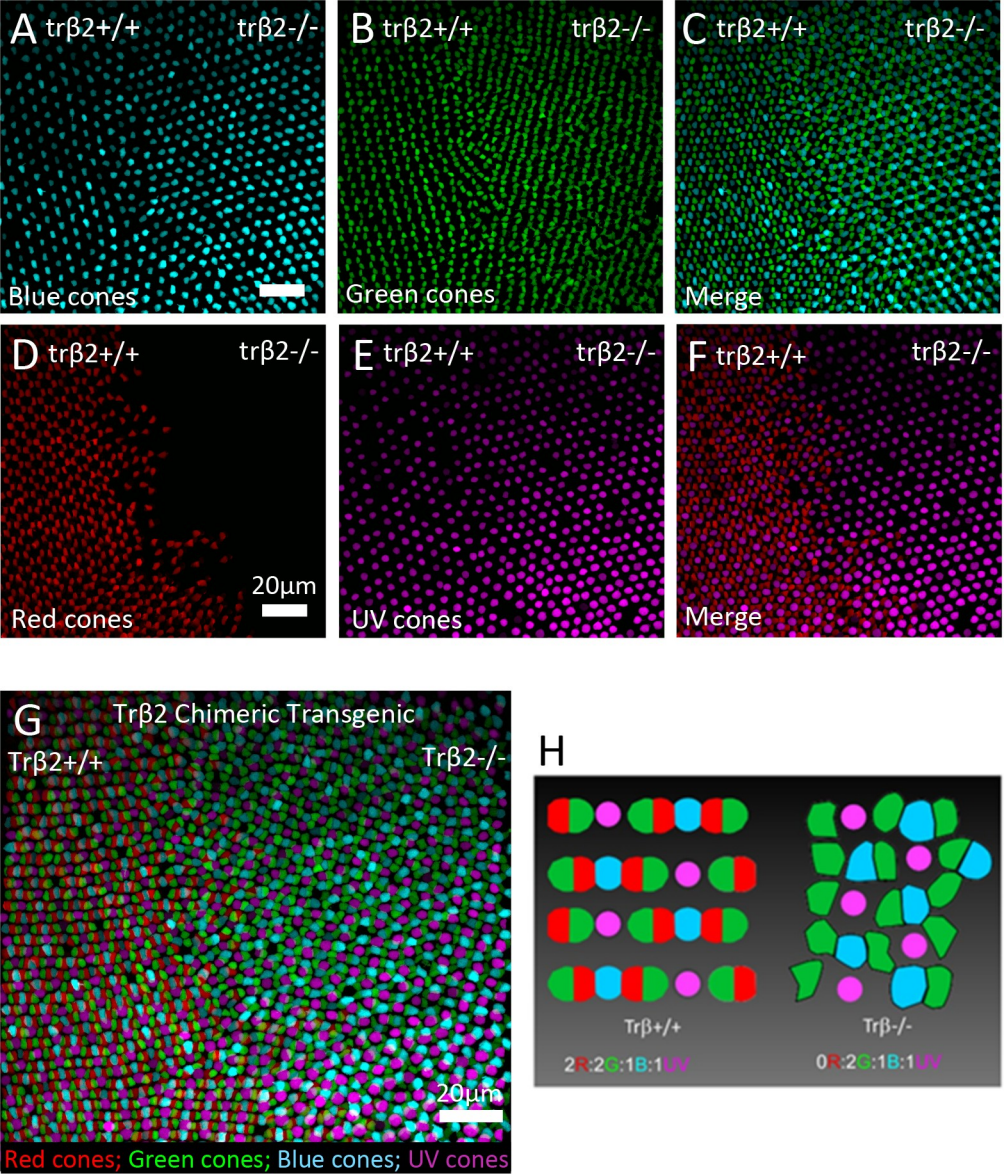
Mosaic analysis of cone photoreceptor spatial arrangement with CRISPR/cas9-mediated genome editing of *trβ2*. Left side of each image is either wild type or *trβ2+/-*. Right side of each image is *trβ2-/-*. Images show patterning of cone types separately (A, B, D, E) and merged in pairs (C, F). (G) All of the cone types are merged in this image. (H) Cone mosaic patterns in wild type/*trβ2+/+* (left) or *trβ2-/-* (right). Red cones (red), green cones (green), blue cones (blue), UV cones (magenta). Scale is the same for each image. The retina is from a 21-day fish, with the embryo injected with CRISPR/Cas9 on day 0. Images are from the same retinal location on one retina. N = 1.

In a CRISPR/Cas9-generated *trβ2* chimeric there is a distinct loss of the *trβ2*:*tdTomato* fluorescent marker for red cones on the right side of the image (Fig 5D). This is indicative of a *trβ2-/-* region. Green and blue cone patterning is altered in this mutant region as these cones appear in close proximity (Fig 5C and 5H), while in the wild type region, blue and green cones are always separated by a red cone (Fig 5G and 5H) [17]. Lacking the principal red member of red-green double cones packed on alternating sides, the green cone column straightens in the mutant region suggesting that the double cone may not exist in the mutant. Cell counts were taken from five 45μm by 45μm sections in both regions of the chimeric retina and averaged. Green cones increased from 72.60 ± 3.53 to 91.20 ± 2.20 (*t*-test, *df* = 8, *P* = 0.0021), blue cones increased from 35.80 ± 1.32 to 49.20 ± 0.49 (*t*-test, *df* = 8, *P* < 0.0001), and UV cones increased from 35.60 ± 2.21 to 48.80 ± 1.02 (*t*-test, *df* = 8, *P* = 0.0006). The total density of cones in the wild type is 215.80 ± 10.27, which is significantly higher than in the mutant (189.2 ± 2.7; *t*-test, *df* = 8, *P* = 0.04). However, the density of the green, blue, and UV cones in the mutant is significantly greater than the density of the green, blue, and UV cones in the wild type (144.0 ± 6.9; *t*-test, *df* = 8, *P* = 0.0003). The mosaic ratio of the *trβ2+/+* region is consistent with previous findings of 2:2:1:1 (R:G:B:UV) in wild type adults [17]. While the ratio between the green, blue, and UV cones remain the same in the mutant (2:1:1), there is a significant increase in the number of each of those cone types (Fig 5). There is still a blue/UV cone column, a green cone column, and a cone mosaic observed in the mutant, though they are not as clearly organized as in the wild type with the red cones present (Fig 5H).

### *6BP+1 trβ2* mutant larvae altered physiology and fractional cone contributions

**Fig 6:**
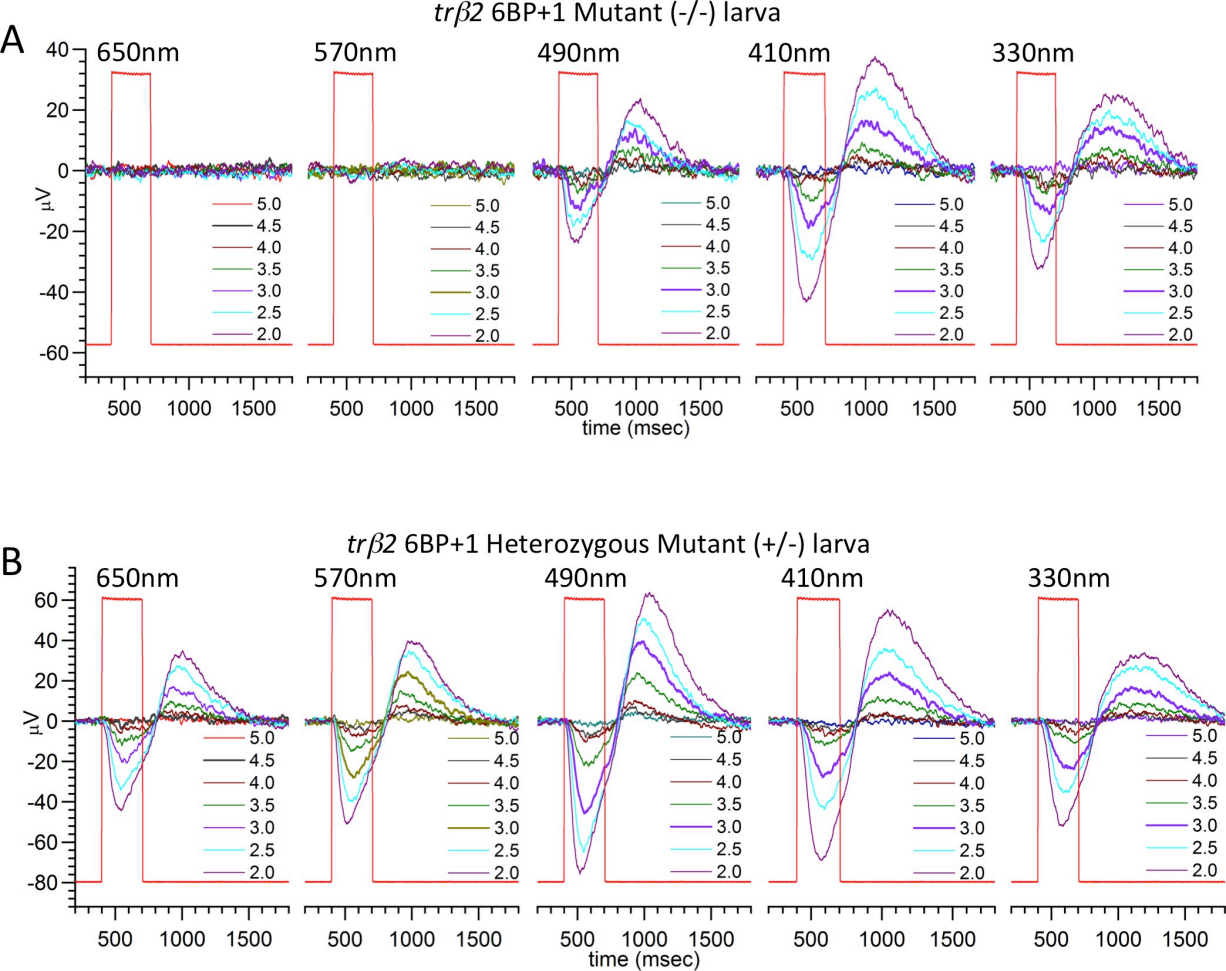
*6BP+1* larval cone PIII ERG spectral response traces. (A) The *6BP+1* mutant does not respond to 650 nm and 570 nm while maintaining a response to shorter wavelengths (N = 12, 70-point datasets). (B) The heterozygote shows sustained responses across all wavelengths (650–330 nm) (N = 16, 70-point datasets). Only half of the spectral dataset is shown in A and B. Wavelength brightness is given in log units of stimulus attenuation, with 5.0 log units being the dimmest stimulus corresponding to the lowest amplitude response, and 2.0 log units being the brightest stimulus corresponding to the largest amplitude response. Larvae are 5 dpf. Cone PIII responses are isolated with 20 mM Na Aspartate.

We blocked signals downstream of the cones to extract the larval cone PIII waveforms. These signals in response to spectral stimulation for a *trβ2 6BP+1* mutant eye (-/-) and a *trβ2 6BP+1* heterozygous mutant eye (+/-) are shown in Fig 6. Each set of 7 nested traces is an irradiance series, with 0.5 log unit increments in stimulus brightness, and with wavelengths as shown for each set delivered consecutively in left to right time order. The cone PIII waveform consists of vitreal hyperpolarization during the light stimulus (rectangular trace) with a vitreal depolarizing rebound peak following stimulus offset. No b-waves appear in these records, as the activity of synapses on second order retinal neurons is blocked by 20 mM Aspartate.

**Fig 7:**
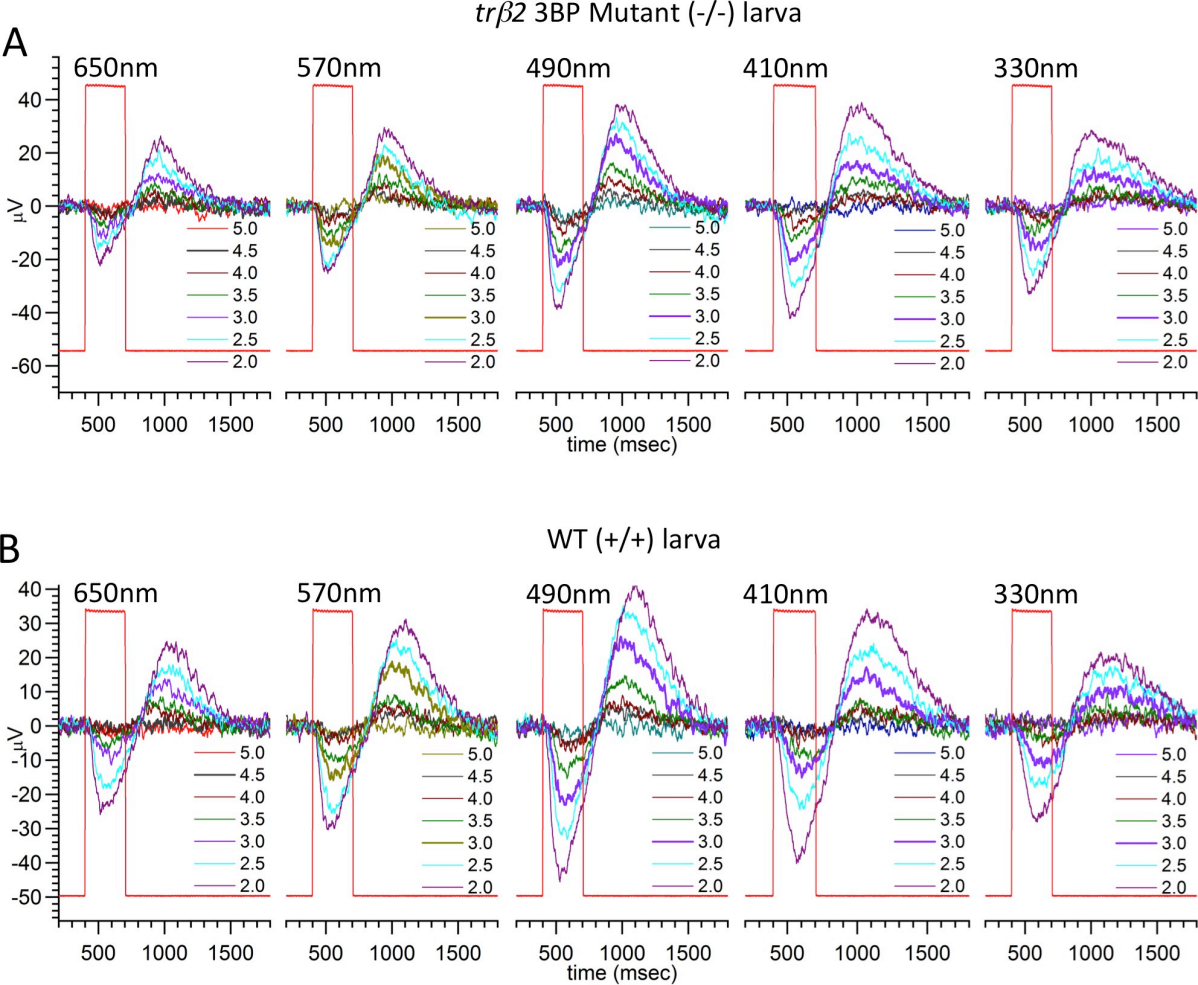
*3BP* larval cone PIII ERG spectral response traces. (A) The *3BP* mutant larva (N = 17, 70-point spectral dataset) responds to all wavelengths from 650–330 nm, with no significant differences from the wild type control (N = 17, 70-point spectral datasets) (B). Larvae are 5 dpf.

In the *trβ2 6BP+1* -/- eye (Fig 6A), PIII ERG responses are only seen for wavelengths shorter than 570 nm. The *trβ2 6BP+1* +/- eye responds at all wavelengths (Fig 6B). This suggests the absence of red (LWS) cone signals in the mutant, but not the heterozygous mutant eye. In a larval *trβ2 3BP* -/- eye (Fig 7A), light responses appear at all stimulus wavelengths, as they do in a wild type (WT) eye (Fig 7B). At wavelengths where responses are evoked, cone signals in all larvae are similar in time course, suggesting little alteration in cone phototransduction kinetics is caused by these mutations.

**Fig 8:**
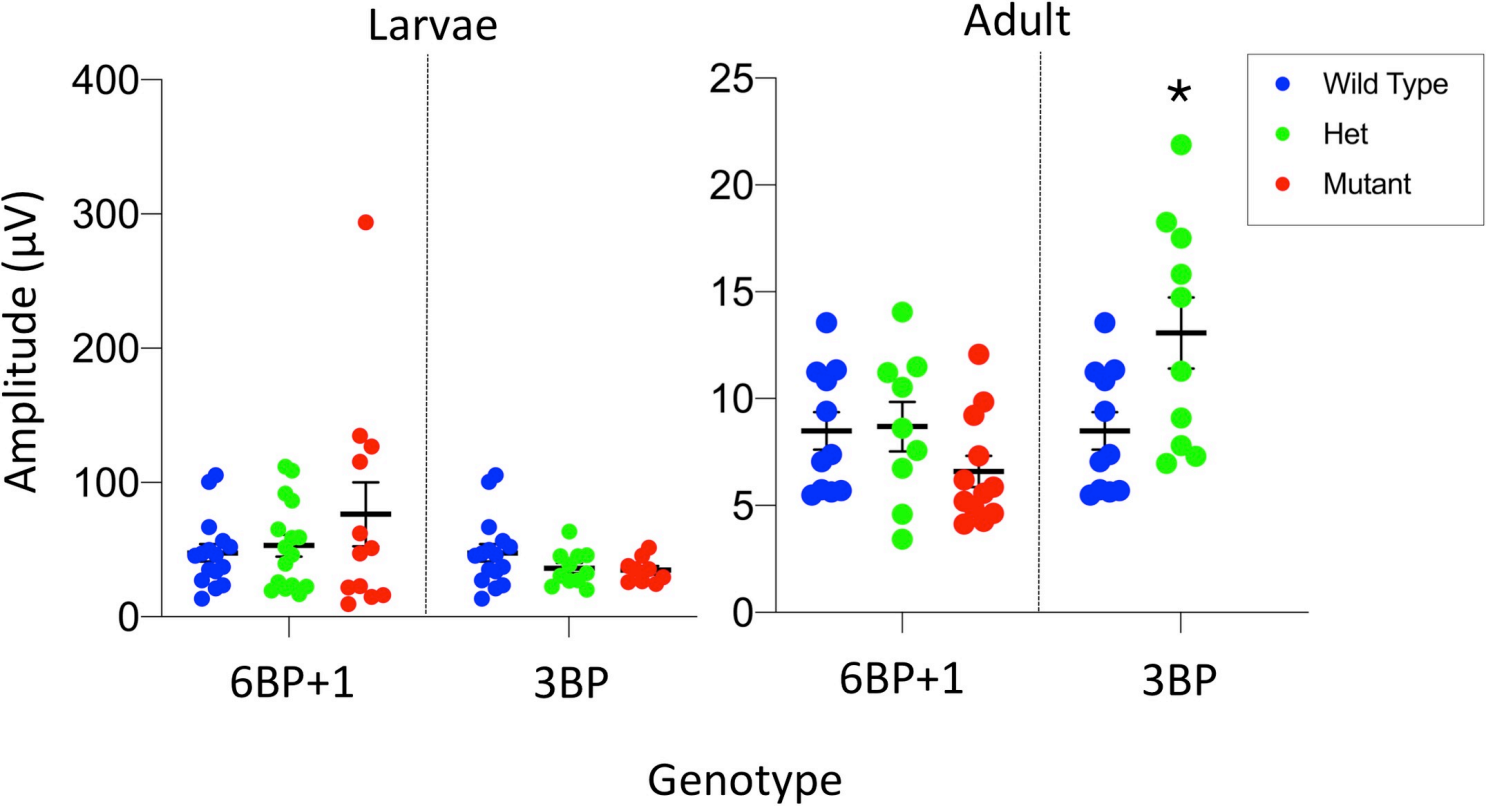
Maximal dataset amplitudes in *trβ2* genetic strains. The maximum cone PIII amplitudes for datasets from each genotype are compared. There is no significant difference between the *6BP+1* 5–7 dpf mutants (N = 12), het mutants (N = 16), and WT (N = 17); nor is there a significant difference between the *3BP* mutants (N = 17), het mutants (N = 33), and WT (N = 17). The *6BP+1* adult mutants (N = 12), het mutants (N = 9) and WT (N = 11) were not significantly different. There was a significant difference (One-way ANOVA, *P =* 0.02) between the *3BP* adult het mutants (N = 10) and wild types (N = 11). There are no *3BP* adult mutants. The wild type groups combine fish from *6BP+1* and *3BP* heterozygote in-cross spawns.

One potential action of a retinal mutation is to change the maximal voltage amplitudes of PIII ERGs. Fig 8 gives the distributions, means and SEs of maximal dataset amplitudes in genetic groupings for the *trβ2* gene in 5–7 dpf larvae and 8–18 month adults. For each mutation, larvae are from the same set of spawns so that other genetic differences are reduced. While there is a trend towards higher amplitude responses in the *6BP+1* mutants and heterozygous mutants, the trend did not achieve significance [ANOVA, F(2, 42) = 1.16, *P* = 0.32]. No amplitude changes were noted among the *3BP* strains [ANOVA, F(2, 61) = 1.80, *P* = 0.17]. Since amplitude changes are not significant, datasets from individual larvae within the genetic groups were normalized to the dataset’s maximal amplitudes and combined into genotype-level datasets for fitting to the spectral model. The number of amplitude-irradiance-wavelength points in these genotype-level datasets varied from 630 for the *3BP* mutants to 1190 for the combined WT group. As larvae were sequenced after physiological recording, there was no prior knowledge of genotype, so it was not possible to match the number of larvae for each genotype-level dataset.

The *3BP* strain appears to be embryonic lethal. Among 56 adults raised from heterozygous *3BP* in-crosses, no adult homozygous mutants were found. Therefore, the lethality could be caused by the *3BP* mutation itself, a closely linked off-target CRISPR hit, or a perturbation of a neighboring gene. Otherwise we would expect to see a *3BP* homozygous mutant adult.

**Fig 9:**
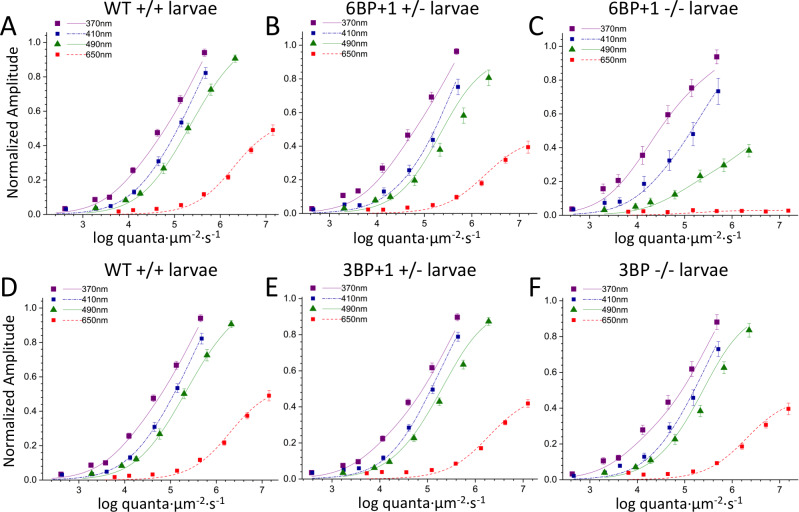
Irradiance plots: Change in amplitude of arval cone PIII ERG responses as a function of brightness and wavelength. (A, B) The wild type (N = 17 datasets, 1190 points total) and *6BP+1* heterozygote (N = 16 datasets, 1120 points total) irradiance-amplitude curves are similar at 4 modeled wavelengths, while the *6BP+1* mutant (N = 12 datasets, 840 points total) (C) shows a loss of response to 650 nm that does not change even at higher irradiance levels. (D-F) Wild type (same as in A), *3BP* heterozygote (N = 33 datasets, 2310 points total), and *3BP* mutant (N = 17 datasets, 1190 points total) larvae are similar in their respective irradiance plots.

Model fits for irradiance response functions in 6 genotype-level datasets are seen in Fig 9. Points are means and SEs from within the combined genotype-level datasets, while the curves result from a model fit (Eq 1) to each genotype-level dataset. These are not individual-cone-type irradiance response points and curves, but the summation of multiple cone signals at each wavelength. The model curves fit all the six different larval genetic datasets well. The most severely altered irradiance response patterns arise in the *6BP+1* -/- mutant (Fig 9C). Responses to red 650 nm wavelengths remain at near ‘0’ amplitude, regardless of brightness. At a normalized amplitude of 0.2, the mutant 490 nm curve is shifted 0.6 log units to the right, towards lower sensitivity, and the mutant 370 nm curve is displaced 0.4 log units left, towards higher sensitivity as compared to WT (Fig 9A and 9C).

The loss of photon sensitivity at 490 nm may not be caused solely by the loss of red cones. Red and green cones are known to be equal in number in the wild type [18]. Since the 460 nm larval green cone opsin and the 556 nm red cone opsin absorb equally at 490 nm, a factor of 2 decrease in sensitivity could be attributed to the red-cone loss in the mutant. The log of 2 is 0.3 log units, suggesting the extra 0.3 log units decrease in the mutant might be attributed to the decreased sensitivity of green cones. This is corroborated by a modeled increase in semi-saturation irradiance in the mutant (4.93 ± 0.13 log(quanta·μm^-2^·s^-1^)) compared to the wild type (4.57 ± 0.08 log(quanta·μm^-2^·s^-1^)) (t-test, *df* = 2028, *P* < 0.05). The irradiance response patterns for the larval *3BP* homozygous mutant (Fig 9F) and heterozygous mutant (Fig 9E) are qualitatively like those of WT larvae (Fig 9D).

**Fig 10:**
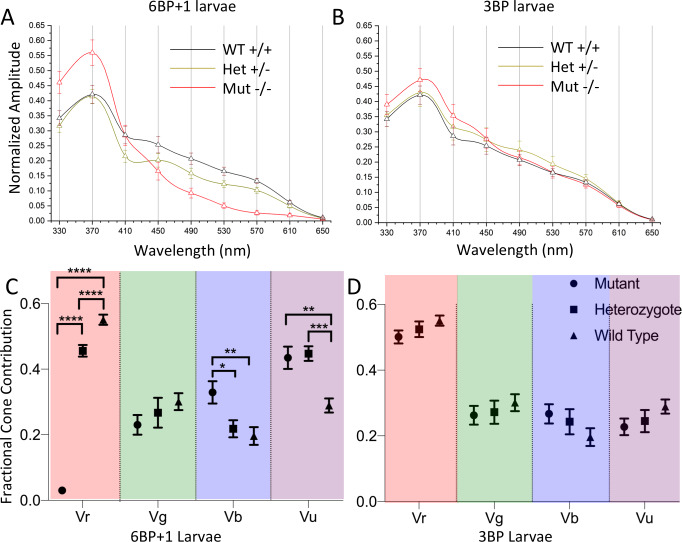
Spectral plots and cone contribution comparisons for *6BP+1* and *3BP* larva. (A) Scanning from 330 nm to 650 nm with constant quantal irradiance (4.6 log(quanta∙μm^−2^∙s^−1^) the *6BP+1* mutant (N = 12) shows a large drop in response amplitude for long wavelengths and a strong preference for UV wavelengths compared to the wild type (N = 17) and heterozygote (N = 16). (B) The *3BP* spectral plot shows a similar spectral pattern in response amplitude across the wavelengths between all genotypes. (C) The *6BP+1* mutant displays a significant drop in red cone contribution (Vr) accompanied by an increase in UV cone contribution (Vu). (D) The cone contribution comparisons between the *3BP* genotypes show a trend towards decreasing Vr for the mutant and heterozygote as compared to wild type, though not significant. Spectral curves and fractional cone amplitudes in A-D are generated from the same datasets and model as in Fig 9.

Larval spectral curves, which are the constant irradiance amplitudes at specific wavelengths normalized relative to the maximal amplitude for all wavelengths and irradiances, are model generated in Fig 10. Response amplitudes are calculated for a constant quantal level of 4.6 log(quanta·μm^-2^·s^-1^), which was below semi-saturation for all but UV cone types. In the *6BP+1* mutant fish (Fig 10A) amplitudes of response are greater than WT for wavelengths shorter than 410 nm, and less than WT for wavelengths longer than 410 nm. In the *3BP*, p.Tyr61del larvae, spectral curves are remarkably similar for mutants, heterozygotes and WT (Fig 10B).

### Cone-signal phenotype of *6BP+1 trβ2* mutant larvae

The goal of the model is to rationalize spectral datasets in terms of changes in three parameters of responsiveness for each of four cones of different spectral type. *Vmax*, the cone saturation amplitude, is a model-fit index proportional to the number of cones and their response voltage as individuals. *Vmax* values for red, green, blue, and UV cones (Vr, Vg, Vb, and Vu) are compared in Fig 10C and 10D. As expected from the above results, the red-cone amplitudes (Vr) in the *6BP+1* -/- mutant were eliminated (Fig 10C) [ANOVA, F(2, 3147) = 281.1, *P* = 0.0000]. The red-cone fractional contribution in the mutant (0.030 ± 0.008) was significantly decreased in comparison to both WT (0.551 ± 0.016) and heterozygous mutant (0.456 ± 0.018) (Fig 10A, Tukey post hocs, *P* = 0.0000). In addition, there was a significant red-cone amplitude-reduction phenotype for the heterozygous mutant in respect to WT (Tukey post hoc, *P* < 0.05). Among the *6BP+1* larval siblings, the three genetic outcomes of the spawns showed UV cone (Vu) amplitudes that were significantly impacted [ANOVA, F(2, 3147) = 13.1, *P* = 0.0000]. There were increased amplitudes of UV cone signals in both mutant and heterozygous mutant larvae as compared to WT (Tukey post hocs, *P* < 0.001, *P* = 0.0000, respectively, Fig 10A). The fractional contributions were 0.289 ± 0.022 (WT), 0.447 ± 0.022 (heterozygous mutant), and 0.435 ± 0.034 (mutant). Additionally, the fractional blue cone contribution was significantly increased in the mutant (0.329 ± 0.034) in comparison to heterozygous mutants (0.218 ± 0.026) and WT (0.196 ± 0.027) [ANOVA: Vb, F(2, 3147) = 5.53, *P* = 0.004; Tukey post hocs, *P* = 0.023, *P* = 0.004, respectively, Fig 10C]. There was a trend towards reduction of fractional green-cone contributions, but no significant differences among the larval sibling genetic types were found [ANOVA: Vg, F(2, 3147) = 0.94, *P* = 0.39]. Results suggest a significant role for the *trβ2* gene in the apportionment of signal strength particularly among red and UV cones.

### Cone-signal phenotype of *3BP trβ2* mutant larvae

The irradiance-response (Fig 9D–9F) and spectral plots (Fig 10B) for the *3BP* deletion mutant do not suggest a spectral phenotype, and the modeled larval cone component analysis indicates only a subtle change. For the *3BP* deletion mutant, fractional red-cone amplitudes (0.501 ± 0.020, Vr) trended towards a reduction in comparison to the WT (0.551 ± 0.016), but this was not significant [ANOVA, F(2, 2587) = 1.63, Tukey post hocs, *P* = 0.18]. None of the other cone types among the *3BP* WT, heterozygous mutant, and mutant larvae varied significantly [ANOVA: Vg, F(2, 2587) = 0.48, *P* = 0.619; Vb, F(2, 2587) = 1.35, *P* = 0.260; Vu, F(2, 2587) = 1.51, *P* = 0.221]. The *3BP* protein appears competent in directing progenitors into the red-cone pool.

### Cone sensitivity peaks in larval *trβ2* genetic strains

The trβ2 nuclear receptor is a transcription factor, and a candidate molecule to regulate cone-opsin expression. For this reason, we compare the fit values for cone sensitivity peaks in the *6BP+1* and *3BP* mutants or heterozygous mutants to WT siblings (Table 1). In no case where a red-cone sensitivity peak could be detected was it significantly changed by either mutant or heterozygous mutant genetics. All peaks lay in a tight range between 550 and 557 nm, similar to previous reports for WT larvae, and consistent with *LWS2* expression [18]. The missing peak, of course, was the *6BP+1* mutant, where there is no red-cone signal from either *LWS2* or *LWS1*. In the *6BP+1* mutant and heterozygous mutants green, blue and UV cone peaks were unaffected (Table 1) and in a range reported for WT larvae [18], corresponding to G1 (*RH2-1*) for green cones, B1 (*SWS2*) for blue cones, and U (*SWS1*) for UV cones. Because of low signal amplitude in mutant larvae, no green-cone opsin peak was fit, however the presence of a green cone amplitude (Vg, Fig 10C) indicates that green cones are physiologically active.

**Table 1:** Peak sensitivities of larval cones in *trβ2* genetic strains.

Larval *trβ2* strain	Cone type	Units	WT (+/+)	Het mutant (+/-)	Mutant (-/-)	*P*
6 BP+1	red (LWS)	nm	553.7 ± 1.8 (1190)	556.7 ± 2.8 (1120)	no fit	0.36
	green (Rh2)	nm	461.8 ± 3.9 (1190)	466.4 ± 4.3 (1120)	no fit	0.42
	blue (SWS2)	nm	403.2 ± 5.5 (1190)	396.4 ± 5.2 (1120)	399.7 ± 4.4 (840)	0.63
	UV (SWS1)	nm	357.5 ± 2.3 (1190)	350.3 ± 3.1 (1120)	354.6 ± 2.7 (840)	0.15
3 BP	red (LWS)	nm	553.7 ± 1.8 (1190)	550.9 ± 2.8 (770)	552.9 ± 2.6 (630)	0.66
	green (Rh2)	nm	461.8 ± 3.9 (1190)	460.2 ± 5.6 (770)	460.6 ± 4.9 (630)	0.97
	blue (SWS2)	nm	403.2 ± 5.5 (1190)	399.9 ± 7.1 (770)	396.8 ± 4.7 (630)	0.76
	UV (SWS1)	nm	357.5 ± 2.3 (1190)	353.0 ± 4.1 (770)	350.1 ± 3.6 (630)	0.24

Larval cone wavelength peaks are fit to genotype-level datasets using Eq 1. Values are wavelength peaks ± SEs (N), where N is the number of amplitude-irradiance-wavelength points fit in each genotype-level dataset.

### *6BP+1* mutant optomotor response

**Fig 11:**
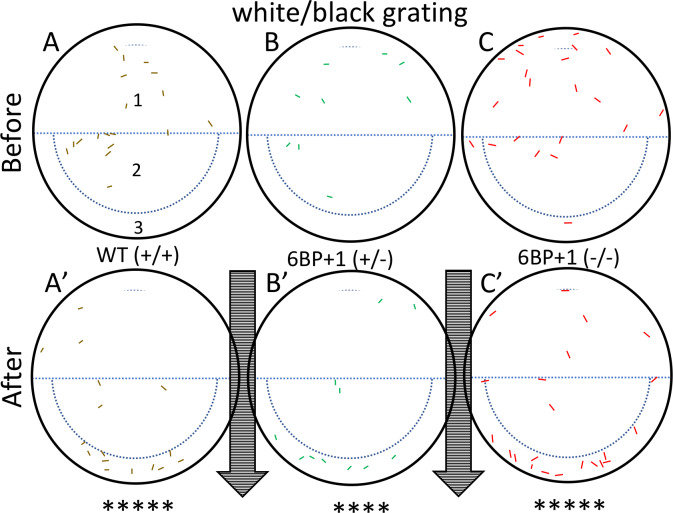
Optomotor response test using a black/white stimulus. Controls use a drifting black and white grating pattern with wild types (+/+) (N = 18 larvae), *6BP+1* heterozygotes (+/-) (N = 12 larvae), and *6BP+1* mutants (-/-) (N = 23 larvae) at 7 dpf. Colored markings in the dishes represent larval zebrafish locations before and after the one-minute stimulus. The arrows indicate the direction of the stimulus. (A, B, C) Wild types, heterozygotes, and mutants respectively start scattered in the dish. (A’, B’, C’) Each genotype moved with the stimulus showing significant pooling of larvae at the bottom edge. Experiment was repeated 2 times per genotype, with similar outcomes for each trial. Significance, given by the Fisher Exact Test, refers to the combined numbers from the two plates (Table 2).

Zebrafish larvae tend to swim in the direction of a drifting grating. This is called the optomotor response (OMR). The response is reported to be driven by both red and green sensing cones in larvae [19], but dependent on red cones in adults [20]. We tested whether this behavior was lost in *6BP+1 trβ2* mutants. Larvae of different genotypes were placed in a tissue-culture plate atop a laptop monitor, where the black/white grating was presented for one minute. The plate was divided into 3 regions (Fig 11A) and the number of larvae in regions 1 and 3 were compared before and after stimulation. For WT larvae (Fig 11A and 11A’), there were 9 larvae in region 1, the top half of the plate, before the stimulus, but only 3 afterwards. In region 3, the bottom rim of the plate, there were 0 larvae before the stimulus, but 10 larvae after the stimulus, showing movement of larvae during the stimulus. The data from two trials were combined for analysis and larval distributions in the plate regions before and after the stimulus are written in Table 2. The change in distribution for wild types is significant (Fisher Exact Test, N = 36, *P* < 0.0001). Both *6BP+1* heterozygous (Fig 11B and 11B’) and homozygous mutant (Fig 11C and 11C’) genotypes showed significant OMR behavior (Fisher Exact Test, +/-, N = 24, *P* < 0.0001; -/-, N = 46, *P* < 0.0001) to the black/white grating. The *6BP+1* mutants are capable of vigorous OMR visual behavior even in the absence of physiological signals from red cones.

**Table 2:** OMR larval distributions before and after white/black stimulus.

Region	WT (+/+)		6BP+1 (+/-)		6BP+1 (-/-)	
	Before	After	Before	After	Before	After
1 and 2	33	14	23	10	42	19
3	3	22	1	14	4	27

Distribution of larvae in culture-plate regions before and after stimulation with white/black drifting gratings.

**Fig 12:**
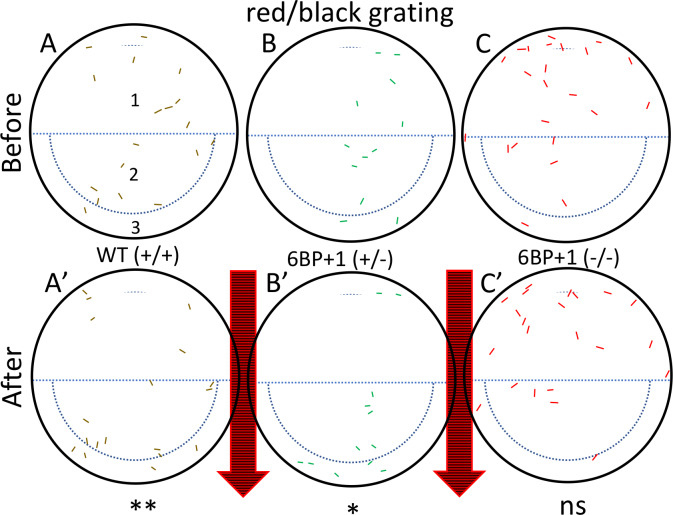
Optomotor response test using a red and black stimulus. The wild type (N = 18 larvae) (A, A’) and heterozygote (N = 12 larvae) (B, B’) larva moved with the stimulus as seen in the white/black drifting gratings (Fig 11). (C, C’) The mutant larvae (N = 23 larvae) did not move with the red/black stimulus and remained scattered. The experiment was repeated 2 times, with similar outcomes in each trial. Significance is calculated with the Fisher Exact Test using data from both trials (Table 3).

Because of a lack of LWS cone physiology, OMR responses to red/black drifting gratings were not expected in *6BP+1* homozygous mutants, and in fact were not found (Fig 12). Of 16 larvae in region 1 of the mutant plate (Fig 12C) 16 remained after stimulation with a red/black grating (Fig 12C’). In region 3, there was 1 larva before and after stimulation. The combined data from both trials are consistent with a lack of mutant OMR for red/black gratings (Fisher Exact Test, N = 46, *P* > 0.99). WT and heterozygous mutant larvae (Fig 12A, 12A’, 12B and 12B’) showed significant OMR behavior under the same conditions (Fisher Exact Test, +/+, N = 36, *P* < 0.01; +/-, N = 24, *P* < 0.05). The combined distributions are shown in Table 3. These genotypes show that red-cone signals alone are sufficient for OMR in wildtype and *6BP+1* heterozygote fish, but the absence of red cones prevents a response in the *6BP+1* mutant fish.

**Table 3:** OMR larval distributions before and after red/black stimulus.

Region	WT (+/+)		*6BP+1* (+/-)		*6BP+1* (-/-)	
	Before	After	Before	After	Before	After
1 and 2	30	17	20	11	44	43
3	6	19	4	13	2	3

Distribution of larvae in culture-plate regions before and after stimulation with red/black drifting gratings.

### *6BP+1* mutant optokinetic response

**Fig 13:**
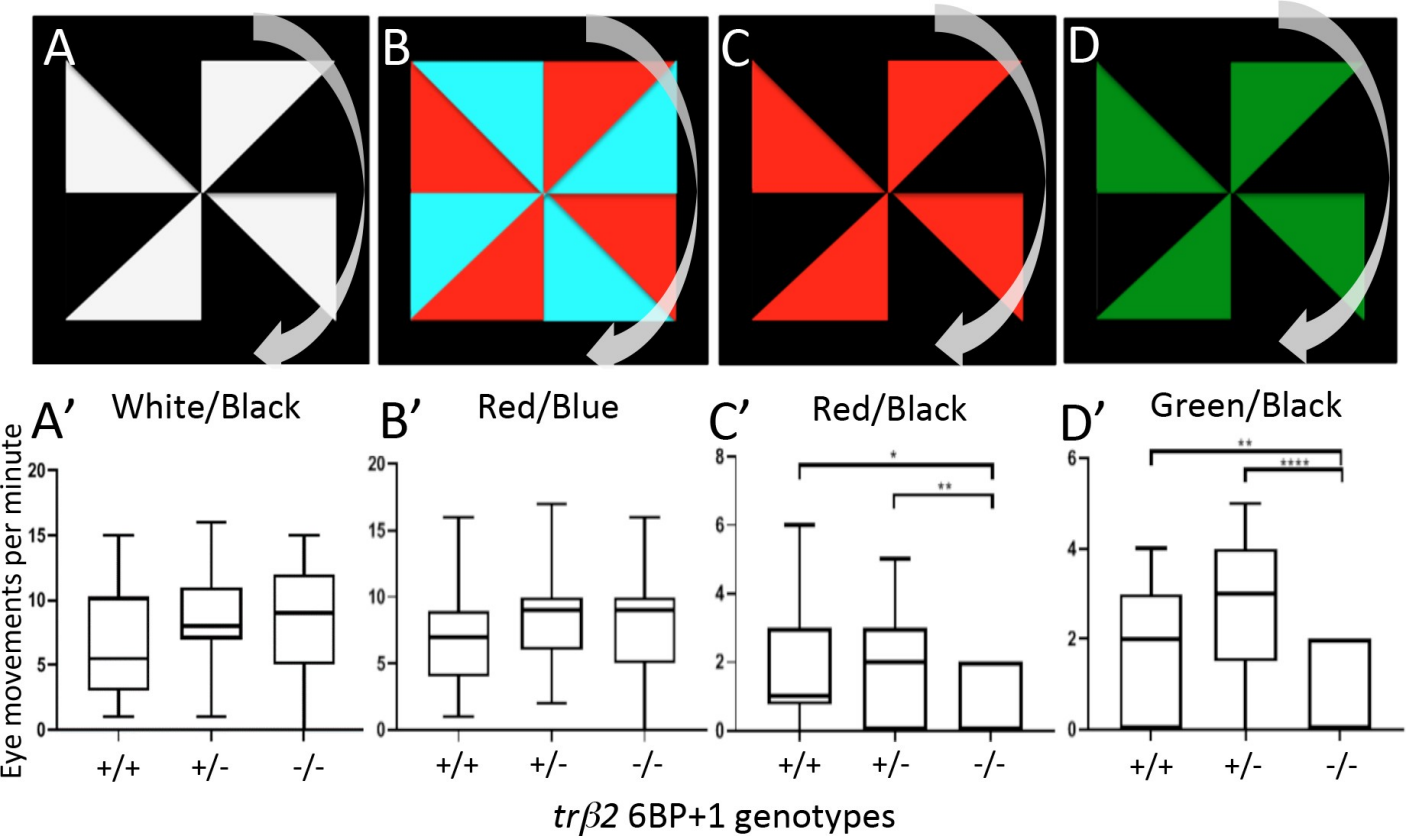
Optokinetic response tests. (A, B, C, D) Each larva at 9 dpf was exposed to these four stimuli in left to right order. The pinwheel patterns moved clockwise. (A’, B’) The wild type, *6BP+1* heterozygote, and *6BP+1* mutant responded similarly to the white/black and red/blue stimuli. (C’, D’) The mutant had significantly less eye movements in response to the red/black and green/black stimuli. There was one repetition for each wild type (N = 30), heterozygote (N = 37), and mutant (N = 19) larvae. Monitor peak wavelengths are red (~610 nm), green (~550 nm), and blue (~450 nm). Significance is calculated with Mann-Whitney U test.

Larval zebrafish eyes track rotating patterns, and then snap back to reset [21]. This optokinetic response (OKR) is an obligatory visual reflex among vertebrates. The question is the role of red cones in this reflex. In Fig 13 eye movements are counted for colored rotating windmills presented on a laptop monitor for one minute. Larvae with mutant *6BP+1 trβ2* homozygous (-/-) alleles, WT (+/+), or heterozygous mutant alleles (+/-) were shown white/black, red/blue, red/black, and green/black pinwheels (Fig 13A, 13A’, 13B, 13B’, 13C, 13C’, 13D and 13D’). The *6BP+1* homozygous mutant fish (-/-) show OKR responses for white/black and red/blue pinwheels, but not red/black (~610 nm) or green/black (~550 nm) pinwheels. The mutant red/black OKR responses (Mdn = 0.0) were significantly lower than WT (Mdn = 1.0) (*Mann Whitney*, U = 188, *P* < 0.05) and the heterozygous mutant (Mdn = 2.0) (*Mann Whitney*, U = 197, *P* < 0.01, Fig 13C’). There was a significant decrease in the number of green/black OKRs between the homozygous mutant fish (Mdn = 0.0) and WT (Mdn = 2.0) (*Mann Whitney*, U = 168, *P* < 0.01) or heterozygous mutant (Mdn = 3.0) (*Mann Whitney*, U = 118, *P* < 0.0001. Fig 13D’). In respect to OKR behavior, the *6BP+1 trβ2* mutant fish do not respond to the monitor colors red (~610 nm) or green (~550 nm). Both colors selectively stimulate zebrafish red cones. This suggests the *6BP+1* mutants, lacking red cones, can use shorter-wavelength cones for OKR given the positive responses to the red/blue pinwheel stimulus. The blue stimulus (~450 nm peak) significantly overlaps with the 460 nm peaking larval green cones.

### Loss of red response in *6BP+1* mutant adult zebrafish

**Fig 14:**
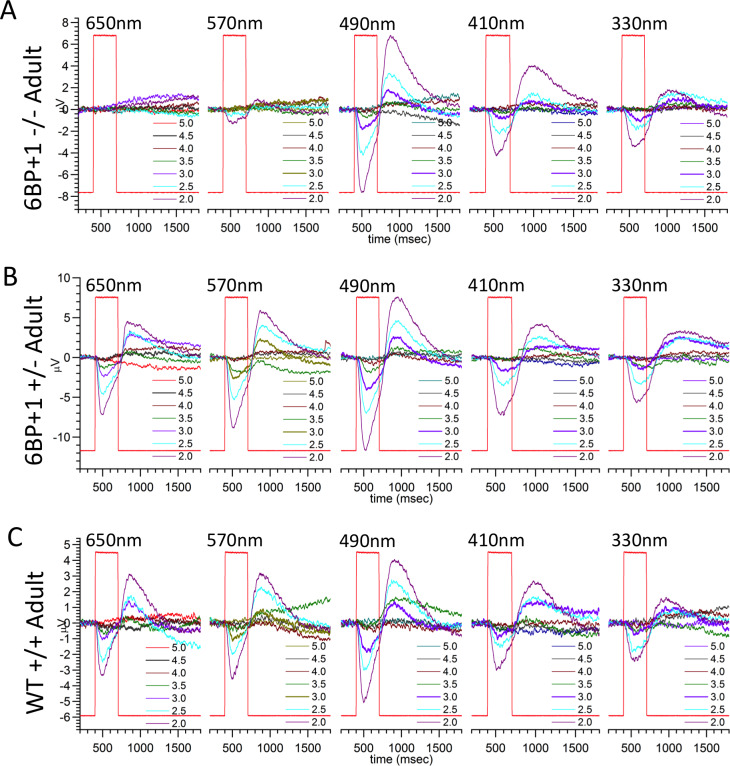
Adult PIII ERG response plots. (A) *6BP+1* mutant adults (7 eyecups, 12 datasets) did not respond to 650 nm, but did respond to the maximum brightness level of 570 nm. (B) Heterozygotes respond to every wavelength (6 eyecups, 9 datasets). (C) Wild types respond to all wavelengths (7 eyecups, 11 datasets). Only half of a dataset is shown for A, B, and C.

As in *6BP+1* homozygous mutant larvae, adult *6BP+1* mutant fish are unresponsive to long wavelength stimuli. In a cone-PIII dataset from an adult *6BP+1* mutant fish (Fig 14A), no cone PIII responses are evoked by 650 nm stimuli of any brightness, whereas WT and heterozygous mutants respond well at multiple brightness’s (Fig 14B and 14C). For the *6BP+1* mutant only the brightest of 570 nm stimuli produces a response, while the WT and *6BP+1* heterozygous adults respond vigorously to multiple stimulus irradiances. For wavelengths eliciting responses, there is little change in response kinetics between the three adult genotypes.

**Fig 15:**
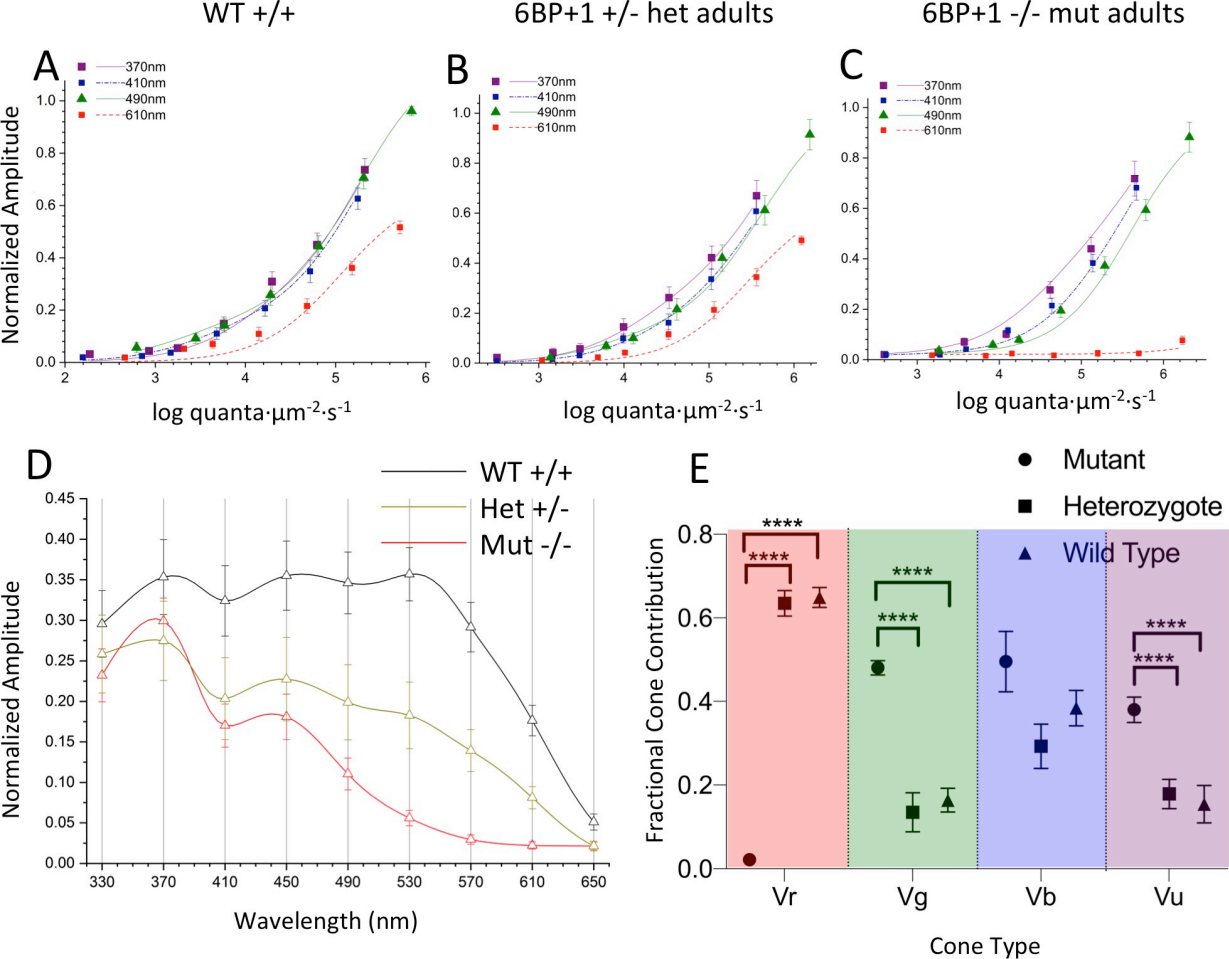
Spectral analysis of *6BP+1* adults. (A, B, C) The irradiance plots for each genotype showed the loss of response to 610 nm in the mutant regardless of brightness. (D) The spectral plot showed a significant loss of amplitude in the heterozygote (N = 9) and mutant (N = 12) at long wavelengths compared to the WT (N = 11). (E) The mutant shows loss of Vr, increase in Vg, and increase in Vu.

For adult genotypes, the genotype-level irradiance-response, spectral, and cone component characteristics are summarized in Fig 15. For WT the model is fit to 770 amplitude-irradiance-wavelength points (7 eyecups), for the *6BP+1* heterozygous mutant, 630 points (6 eyecups) and the *6BP+1* mutant, 840 points (7 eyecups). Dataset maximal amplitudes were not affected by genotype (Fig 8).

The model curves closely fit irradiance response data points at multiple wavelengths in the three genotypes (Fig 15A, 15B and 15C). In the *6BP+1* mutant (Fig 15C), there is a complete loss of responsiveness at 610 nm, whereas WT and heterozygous mutants show increasing response amplitudes with brighter 610-nm irradiances (Fig 15A and 15B). The 370 nm, 410 nm and 490 nm curves are closely bunched for WT but separate for *6BP+1* heterozygotes and especially mutants, indicative of spectral differences. The 370 nm curve shifts 0.2 log units to the left for the heterozygote and 0.4 log units to the left for the mutant in respect to the wild type. Both shifts are suggestive of greater sensitivity for *6BP+1* mutant or heterozygous mutant UV cones, as seen in larvae. The 490 nm curve-shift of 0.3 log units to the right for the mutant and 0.1 long unit to the right for the heterozygous mutant may be attributed to both the loss of red cone signals at 490 nm and an increase in green-cone semi-saturation irradiance. The semi-saturation irradiance for green cones increases from 3.17 ± 0.17 log(quanta·μm^-2^·s^-1^) in wild types and 3.73 ± 0.31 log(quanta·μm^-2^·s^-1^) in heterozygotes to 5.64 ± 0.0.09 log(quanta·μm^-2^·s^-1^) in mutants [ANOVA: F(2, 2237) = 47.9, *P* = 0.0000].

The spectral curves, PIII fractional amplitudes for constant quantal irradiance across the spectrum, show a different spectral phenotype for each genotype (Fig 15D). The stimulus is 4.6 log(quanta·μm^-2^·s^-1^), in the range of semi-saturation for adult cone types. While amplitudes for UV stimulation are similar in WT, *6BP+1* mutant, and heterozygote, the mutant and heterozygote evoke lesser amplitudes at longer wavelengths. In the *6PB+1* mutant, there is no spectral response at this stimulus irradiance for wavelengths longer than 570 nm, and the heterozygote response at these wavelengths is intermediate compared to WT.

In the adult *6BP+1* mutant genotype, red and UV cone saturation amplitudes (Vr, Vu, Fig 15E) are altered in a way similar to *6BP+1* larvae (Fig 10C). Vr is severely diminished in the adult *6BP +1* mutant fish as compared to either WT or heterozygous mutants [ANOVA: F(2, 2237) = 312.8, *P* = 0.0000]. Unlike *6BP+1* mutant larvae, heterozygous adults show no significant diminution in Vr as compared to WT. The loss of *6BP+1* heterozygous long-wavelength spectral amplitude in Fig 15C is caused by an increase in red-cone semi-saturation irradiance from 4.79 ± 0.07 log(quanta·μm^-2^·s^-1^) in wild types to 5.21 ± 0.07 log(quanta·μm^-2^·s^-1^) in heterozygotes (*t*-test, *t* = 4.99, *df* = 1398, *P* < 0.001), a significant desensitization of adult red cones. In the adult *6BP+1* mutant fish, Vu is significantly greater than WT [ANOVA: F(2, 2237) = 11.7, *P* = 0.0000]. Unlike *6BP+1* larvae, the adult Vu value for heterozygotes is not increased, but the same as WT. Similar to larvae, adult blue cone amplitudes (Vb) trend larger in the mutant [ANOVA: F(2, 2237) = 2.87, *P* = 0.057]. The greatest departure from the larval pattern is in the green cones. In addition to the increase in kg in the adult *6BP+1* mutant, Vg amplitude is greatly increased, both in respect to adult WT and to adult heterozygotes [Fig 15E: ANOVA: F(2, 2237) = 13.0, *P* = 0.0000]. The missing Vr amplitude is distributed across Vg, Vb, and Vu in the mutant (Fig 15E). In adults, red cone signals are adversely affected in both mutants and heterozygotes.

### Cone sensitivity peaks in adult *trβ2* genetic strains

We compare the fit values for cone sensitivity peaks in the *6BP+1* and *3BP* mutants or heterozygous mutants to WT siblings (Table 4). In no case where red-cone sensitivity peak could be detected was it significantly changed by the presence of a mutant gene. All peaks lay in a tight range between 566 and 569 nm, similar to previous reports for WT adults, and consistent with adult *LWS1* opsin expression [18]. There are missing peaks in the *6BP+1* and *3BP* mutant, in the first, because of a lack of red-cone signal, and in the second because the homozygous mutation is embryonic lethal. In the *6BP+1* mutants and heterozygous mutants blue and UV-cone peaks were unaffected (Table 4) and in the *3BP* heterozygote, the UV-cone peak was unaffected. In *6BP+1* mutant, the green-cone peak was significantly shifted to longer wavelengths, from 465 ± 6.5 (wild type) and 469.6 ± 11.7 (heterozygote) to 502.2 ± 6.7 (mutant) [ANOVA: F(2, 2237) = 6.468, *P* < 0.01]. This would be consistent with a shift in electrophysiologically active opsin from *RH2-2* to *RH2-4* [11].

**Table 4:** Peak sensitivities of adult cones in *trβ2* genetic strains.

Adult *trβ2* strain	Cone type	Units	WT (+/+)	Het mutant (+/-)	Mutant (-/-)	*P*
6 BP+1	red (LWS)	nm	566.8 ± 3.1 (770)	568.3 ± 4.3 (630)	no fit	0.84
	green (Rh2)	nm	465.6 ± 6.5 (770)	469.6 ± 11.7 (630)	502.2 ± 6.7 (840)	**
	blue (SWS2)	nm	447.9 ± 5.4 (770)	441.4 ± 8.0 (630)	438.4 ± 5.1 (840)	0.51
	UV (SWS1)	nm	364.4 ± 7.4 (770)	351.8 ± 7.2 (630)	359.6 ± 2.1 (840)	0.33
3 BP	red (LWS)	nm	566.8 ± 3.1 (770)	567.3 ± 2.4 (700)	lethal	0.90
	green (Rh2)	nm	465.6 ± 6.5 (770)	487.4 ± 7.3 (700)	lethal	*
	blue (SWS2)	nm	447.9 ± 5.4 (770)	no fit	lethal	-----
	UV (SWS1)	nm	364.4 ± 7.4 (770)	361.4 ± 2.0 (700)	lethal	0.74

Adult cone wavelength peaks are fit to genotype-level datasets using Eq 1. Values are opsin wavelength peaks ± SEs (N), where N is the number of amplitude-irradiance-wavelength points fit in each genotype-level dataset. Probabilities are ANOVAs.

**Fig 16:**
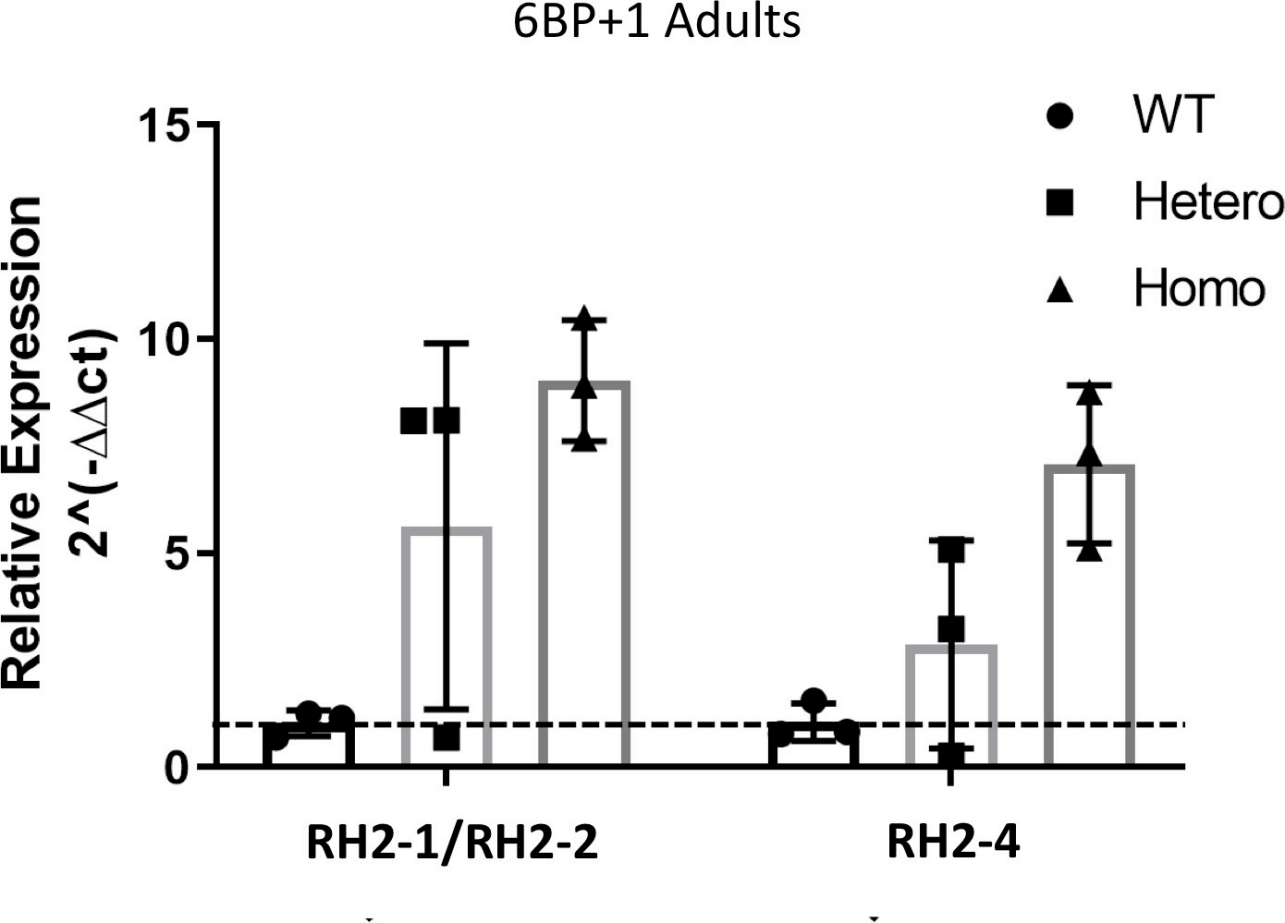
RT-qPCR Analysis. The expression levels of combined *RH2-1/RH2-2* and *RH2-4* were compared between *6BP+*1 wild types (N = 4), heterozygotes (N = 4), and homozygotes (N = 4). The y-axis displays the fold change as compared to wild types, where the mean is set to 1 (dashed line). The x-axis displays the genotypes and opsin types. The expression levels are normalized to the wild type. The experiment was performed twice, each with 3 replicates.

RT-qPCR results in Fig 16 show an increase in both the combined *RH2-1/RH2-2* and *RH2-4* green-cone opsins mRNAs in the *6BP+1* mutant compared to the wild type and heterozygote. Additionally, there is an increase in the heterozygote *RH2-1/RH2-2* and *RH2-*4 expression levels compared to the wild type (Fig 16). These results show that *RH2-4* mRNA does increase its expression, as implied by spectral analysis. The wavelength and sensitivity change of green cones in the *6BP+1* mutant, together with the change in RH2 mRNAs, suggest an alteration of the mutant’s green cones. On this theme, a significant long-wavelength shift of the adult *3BP* heterozygote green cone is also noted (Table 4). There appears to be a long-term role for *trβ2* in green-cone maturation.

## Discussion

Two *trβ2* mutant strains provide insights into the roles of this transcription factor in the vertebrate retina. Both mutations target the same PY amino acid sequence in the N-terminal region of the gene. This is a ‘transactivation’ region, critical to binding other transcription factors and to the activation or inhibition of the transcription process [5]. Both mutants had functional consequences, but the phenotypes differed.

### Frameshift mutant

The loss of response to red wavelengths in the zebrafish *6BP+1 trβ2* mutant is consistent with the losses seen in mouse and human mutants, corroborating the conservation of *trβ2’s* function among vertebrates in mediating the development of long-wavelength cones and long-wavelength sensory pathways [1, 3, 4]. In addition to the significant decrease in responses to red wavelengths, the absence of *trβ2* in zebrafish retina development leads to an increase in UV cone response amplitudes and fractional UV cone contribution. This could either be negative regulation of UV cones specifically by *trβ2*, since thyroid receptors are capable of both positive and negative regulation [5, 22], or a secondary consequence. In larval zebrafish, the significant decrease in Vr and increase in Vu for the *6BP+1* heterozygote in comparison to the wild type identifies a heterozygous phenotype resembling the mutant but to a lesser degree.

While we see a significant decrease in the green-sensitivity in both larval and adult *6BP+1* mutants, we only see a significant increase of Vg and a shift to a longer-wavelength spectral peak for green cones in the *6BP+1* adult mutant. Of the four *RH2* (green opsin) subtypes, *RH2-2* has the highest mRNA expression level in wild type adult zebrafish retina with an in-solution opsin spectral peak of 476 nm [11, 23], and *in situ* spectral measurements corroborate a peak at ~480 nm [18]. In our *6BP+1* mutant adult we see the green opsin spectral peak is fit to 502 nm, which is closely aligned with the *RH2-4* solution peak at 505 nm suggesting the loss of trβ2 consequently induced an increase of *RH2-4* phototransduction [11]. The RT-qPCR results indicated an increase in both the combined *RH2-1/RH2-2* and *RH2-4*, so the preference for RH2-4 opsin is not caused solely by the mRNA expression level. The long-wavelength shift in mutant green-cone opsin provides a partial spectral compensation for red-cone loss.

In development there is a shift from LWS2 to LWS1 opsin [24] and a concomitant shift in physiological red-cone opsin peak from 556 nm to 572 nm [18]. Thyroxin T3 may mediate this shift [25]. While *6BP+1* mutant fish lack LWS opsins altogether, heterozygous mutant fish show a developmental shift in red-cone spectral peak similar to wild type, so that at least modest reductions in trβ2, which are enough to induce a unique spectral phenotype in the heterozygote, do not impede the LWS2-LWS1 opsin shift with development.

In the *6BP+1* mutant fish there is no immunoreactivity for LWS opsins, in either larvae or adults. It also appears there is no immunoreactivity for arrestin (arr3a), as determined with the zpr-1 antibody, raised against mouse arrestin3 and often used to label zebrafish red-green double cones [26]. Zebrafish double cones express arr3a [15]. This suggests *trβ2* is a requirement for the expression of LWS1, LWS2, and arr3a proteins. The transgenic reporter *trβ2*:*tdTomato* used to mark red cones [10] is inactive in the *6BP+1* mutant fish. The *trβ2* promoter used in this transgene would appear also to require the trβ2 transcription factor for activation, evidence that *trβ2* is a self-activating gene. As might be expected of a factor critical to the generation of a neural type, trβ2 activates multiple molecular pathways. Opsin-promoter-based transgenic reporters for green, blue, and UV cones continue to be expressed in the *6BP+1* mutant fish and reveal the distributions of these remaining cone types. The densities of these cone types increased in the mutant, however the total cone density was significantly decreased in comparison to the wild type. It is likely that red cones never develop and those progenitors find other developmental pathways, as we would not see a higher density of the remaining three cone types if red cones died later in development. Additionally, these remaining cones may increase in number with more space, but the lack of double cones decreases packing efficiency, thus somewhat bringing down the total density of cones. Despite changes in cone number, the cone ratios are preserved. Mechanisms for generation of cone density ratios may not require functional red cones, or trβ2.

Arrestin quenches the activated state of opsins. Morpholino suppression of arr3a in zebrafish larvae delays the recovery of b-wave responses to the white stimuli [15]. In *6BP+1* mutant adult fish, altered green-cone signals are observed. One of the changes is decreased sensitivity, as measured by half-saturation irradiance. This might be a consequence of arrestin loss. It is not clear whether the trβ2 influence on arr3a expression in green cones is cell autonomous, or non-cell-autonomous. Several models of cone mosaic formation involve cell-cell interactions among cone progenitors [26, 27]. In studies of *trβ2*:*tdTomato* reporter expression, in situ hybridization only revealed *trβ2* mRNA in cones with reporter fluorescence [10]. Since *trβ2* mRNA is not shown in non-red cones, expression of arr3a in green cones appears to require red cones.

### Tyrosine deletion mutant

While the *3BP* mutant strain did not lose its ability to respond to red wavelengths, the single codon deletion trended towards a decrease in red cone contribution. The Tyr61 deletion located in the N-terminus lies within a potential hormone-independent transactivation domain [5]. Previously it has been noted that patients with resistance to thyroid hormone due to mutations in the *thrb* ligand binding domain lack eye dysfunction [5]. In zebrafish, larvae with ablated thyroid glands maintain the same levels of *LWS2* expression as the controls with a working thyroid gland [25]. These findings suggest that *trβ2* is capable of inducing red-cone development independent of thyroid hormone [5]. Our results are in line with that hypothesis. Although *3BP* mutants still respond to red light, the tendency towards a decrease in cone contribution suggests that this N-terminus located tyrosine is important for the full functionality of *trβ2*. Contrary to our *6BP+1* mutant, the *3BP* mutant did not survive to adulthood, so there does seem to be a consequence of this mutation that is essential for survival [28]. The latest detected developmental stage for the *3BP* mutant is 12 dpf as recordings were conducted at that time. Beyond 12 dpf the mutation appears to be lethal as 0 of the 56 genotyped adult (>3 months post fertilization) offspring from a *3BP* heterozygous breed were mutants. This result suggests further investigation as to why this specific deletion could prove to be lethal, and at what developmental stage does it become lethal, given that the *6BP+1* mutant fish survive to adulthood.

### Behavior

To supplement the ERG results for the frame-shift *trβ2* knockout, the optokinetic and optomotor response behavior tests gave insight into brain function. Wild type fish will swim with the stimulus and congregate at one side of the petri dish [29, 20]. However, the *6BP+1* mutant fish did not follow in suit for the red/black stimulus. The lack of response to the red and black stimulus by the *6BP+1* mutant fish showed that this loss starting in the cones is extended all the way to the brain. The larvae are unable to perceive red hues, thus eliminating their ability to discern red from black; nonetheless the OMR behavior persists using shorter-wavelength cones, most likely the green cones, with the control stimulus. Orger et al [19] suggest both red and green cones contribute to larval optomotor response.

For the optokinetic response, the green light emitted from the monitor peaks at 550 nm [30]. This is closer to the larval red opsin spectral peak of 556 nm than green opsin at 461 nm, so although humans perceive the 550-nm stimulus as green using 535-nm peaking M cones [31] it actually best activates red opsin in larval zebrafish [18]. As a result, we see an even larger significant difference between the genotypes with green/black (~550 nm) stimuli because it excites the red cones in wild types and heterozygotes more than the red stimulus (~610 nm), while maintaining spectral distance from the green cones in the mutants. The control white/black stimulus, as well as the red/blue in the optokinetic experiments, resulted in no differences between the three genotypes, so red cones participate in, but are not vital to the larval OKR pathway provided that other cones are stimulated.

### Summary

Spectral physiology, antibody stains and image analysis of the adult *trβ2* chimera fish confirmed that *trβ2* is required for the differentiation of red cones and LWS opsin in zebrafish [1, 10, 32]. The larval stains of the *6BP+1* mutant showed a complete loss of red opsin, therefore a loss of functional trβ2 results in a loss of red opsin. The adult retina stains confirmed that the loss of red opsin in the mutant is maintained through development, despite the wild-type developmental shift from LWS2 red opsin in larvae to LWS1 red opsin in adults. The adult chimeric further confirmed the mutant loss of red cones as well as changes in mosaic patterning. In adult zebrafish, the cones are arranged in alternating columns of ultraviolet/blue and red/green, which create a mosaic pattern with one UV/blue column for every two columns of red/green [33, 17, 27]. The loss of red cones in the mutant led to significant increases of the other three cone types, but remaining cones maintain their respective mosaic ratios as seen in the wildtype. The images align with the adult cone spectral physiology as there was a significant or trending increase in the green, blue, and UV fractional cone contributions in *6BP+1* mutant adults. In mutant mosaic patterning, unexpectedly, blue cones were in close proximity to green cones when red cones were missing, but the cones still attempt to form alternating blue/UV and green cone columns. This suggests the loss of *trβ2* and subsequently red cones leads to a partial loss of regulation in patterning and abundance of each cone type.

Our studies contribute to the understanding of cone development. We now know that along with *trβ2’s* role in the structural development of the cone layer, there are in fact alterations that extend to cone physiology and zebrafish behavior reflecting what was seen in immunohistochemistry. Future studies will examine *6BP+1* retinal circuitry to investigate downstream changes such as bipolar cell output and horizontal cell regulation. The *3BP* mutant lends itself to investigations on its lethal characteristics and what other pathways could be involved in the first exon of *trβ2*.

## Materials and methods

### Ethics statement

*All procedures for breeding and experimental use were approved by the National Institute of Neurological Disorders and Stroke/National Institute on Deafness and Other Communication Disorders/National Center for Complementary and Integrative Health IACUC (ASP 1307, ASP 1227)*.

### Zebrafish

Zebrafish (*Danio rerio*) (AB strain) were kept in Aquatic Habitats benchtop systems (Pentair Aquatic Eco-Systems). Larvae ages 5–7 days post fertilization (dpf) were kept in an incubator at 28° C in 3.5-inch Petri dishes filled with larval medium. The larval medium was made up of 60 mg/liter sea salt and 75 *μ*l/liter 1.5% methylene blue (Sigma-Aldrich Cat. No. 03978). At 8 dpf, the larvae were transferred to system nursery tanks (520–650 μS water, 28° C, pH 7.5–7.7) and fed Larval AP100 (Pentair Aquatic Eco-Systems) and live rotifers (*Brachionus Plicatilis*, Reed Mariculture) until experimental use at 12 dpf. Adults were kept in the same system environment as the 12 dpf larvae but were fed ground tetramin flakes and live rotifers.

### CRISPR/Cas9 and Phenotyping

Germ-line mutations of *trβ2* were generated by Cas9 mRNA and tr*β*2-gRNA co-injections into the one-cell stage embryos of *Tg(thrb2*:*tdTomato)q22* or *Tg(thrb*:*MA-YFP)q23* according to the method described previously [34]. The targeting sequence is 5’-GGCAACACAGCCAACCCTAT-3’ which resides in the first exon of *trβ2*, the only exon not shared by *trβ1*. Between 12 hpf and 4 dpf the injected embryos were raised in 300 μM Phenylthiourea (PTU, Sigma-Aldrich, catalogue P7629) to block melanin synthesis. At 4dpf, larvae with weak mosaic fluorescent expression of tdTomato or YFP in the photoreceptor layer were screened as potential carriers of germ-line mutations of *trβ2* and raised. The carriers of germ-line mutation of *trβ2* were identified by in-crossing the potential carriers. The rationale was that *trβ2* mutants might have a fluorescence phenotype, as *trβ2* was thought to be a self-activating gene. Suspected mutant lines were in-crossed for multiple generations. To sort the fluorescence phenotypes, larvae were raised in 300 μM Phenylthiourea (PTU, Sigma-Aldrich, catalogue P7629). At the age of 4 dpf larvae were anesthetized in a 3.5-inch Petri dish with ~0.5 ml of Tris buffered 0.4% tricaine in 45 ml of egg water before sorting based on fluorescence in the eye’s photoreceptor layer.

### Mosaic analysis of cone photoreceptor spatial arrangement

Chimera fish that were mosaic of *trβ2+/+*, *trβ2+/-*, and *trβ2-/-* cells were produced by injecting *trβ2* gRNA and cas9 mRNA into 1-cell stage embryos of a quadruple transgenic line, *Tg(gnat2*:*H2ACFP*, *sws1*:*H2AYFP*, *sws2*:*GFP*, *trβ2*:*tdTomato)*. Retinas were fixed at 21 dpf. 3D stack images were acquired using a confocal microscope that was equipped with 440, 515 and 561 nm laser for exciting CFP, YFP and tdTomato, respectively. Image analysis was conducted using Amira. Green cone nuclei were identified by subtracting red-, blue-, and UV-cone fluorescence from the *gnat2*:H2ACFP fluorescence, which marks all four cone types.

### Genotyping

To determine genotyping of the fish we performed sequencing from fish genomic DNA. The fish genomic DNA was extracted from whole larva or adult fish tail fin snip by KAPA express extract kit and PCR was performed using primers that span the target site of interest, the forward primer 5’-CATGGTGTAAGTGGCGGATATG-3’ and the reverse primer 5’-TCCACTGCATCTGAGAGAAATCC-3’. The PCR primer pairs were designed by the primer3 program (http://primer3.sourceforge.net). PCR reactions were completed in 10 μl volumes containing 40 ng of genomic DNA, 1 μl of the forward and reverse primers at 10 μM, 1 μl of 10XPCR Buffer (100 mM Tris HC1(pH8.4)), 2.5 mM MgCl_2_, 2.5 mM dNTP mix and 0.2 U Taq DNA Polymerase. The thermal cycling conditions were as following: 94°C for 3 min, 35 cycles of 94°C for 30 sec, annealing temperature at 58°C for 30 sec and 72°C for 40 sec, and a final extension at 72°C for 3 min. PCR products were purified by using the AMPure XP system (Beckman Coulter, Biomek NX). Genomic PCR products were sequenced, the PCR primers were used for bidirectional sequencing using Big Dye Terminator Ready reaction mix according to manufacturer instructions (Applied Biosystems). Sequencing products were purified using the Agencourt CleanSEQ system on a Beckman Coulter, Biomek NX. Sequencing was performed on an ABI PRISM 3130 Automated sequencer (Applied Biosystems) and the sequencing results were analyzed using Mutation Surveyor v3.30 (Soft Genetics Inc., State College PA).

### Isolation and Perfusion of Eyes

Larvae at ages 5, 6, 7, and 12 dpf were isolated on a glass lantern slide, then transferred to a piece of nitrocellulose filter paper (Millipore, 0.45μm pore, Cat. No. HABP02500). With a 37 mm insect pin (Carolina Biological Supply), cuts were made behind the eyes to decapitate the larvae then longitudinally to make a dorsal-ventral cut between the eyes resulting in an isolated eye facing upward. Adults were decapitated with a fresh single-edged razor then longitudinally hemisected between the eyes. Attached tissues were removed from around the eye and the eye was placed upright on a piece of nitrocellulose filter paper. Under a dissecting scope, the cornea was sliced open to remove the lens and open the eyecup. In the recording chamber, the isolated eye was placed in an inverted lid of a 35-mm culture dish (ThermoFisher Scientific), with a disk of 41 μm nylon net filter (Millipore) covering the bottom to wick away perfusate. Larval eyes were perfused at 0.07 ml/min with minimal essential medium (MEM, Thermo FisherScientific, Cat. No. 11090–099, equilibrated with 95% O2, 5% CO2) using a syringe pump (New Era 500L, Braintree Scientific) and a 28-guage microfil syringe needle as an applicator (World Precision Instruments, MF28G67). The microfil applicator was positioned on the nylon mesh. Patch electrodes (3 μm tip) made using a Flaming/Brown microelectrode puller (Model P-87, Sutter Instrument, Novato, CA) and filled with 500 mM NaCl were inserted transcorneally to record the massed cone PIII ERG signals. Adult eyecups were perfused with MEM (as above) at 0.3 ml/min. The perfusion applicator was placed directly in the adult eyecup to ensure retinal oxygenation. Shaved down microelectrodes (300 μm tips), placed in the eyecup, recorded cone-PIII signals [37]. To record cone PIII ERG signals, L-Aspartate (Sigma-Aldrich, catalogue 11195, 20 mM larvae, 10mM adults) was added to MEM perfusate to block post-synaptic, glutamatergic, photoreceptor mechanisms.

### Electroretinogram Physiology

As in Nelson 2019 [18], light stimuli were obtained from a 150W OFR Xenon arc, shutter (Vincent Associates, Cat. No. LS6ZM2, 300 ms steps at 2.5–6.0 sec intervals), interference filters (330–650 nm, 40 nm increments, 20 nm half-width, Chroma Technology), metallic neutral density filters (7.5 log units, 0.5 log unit steps, Andover Corporation), computer-driven filter wheels, and liquid light guide (Sutter instruments, Lambda 10–3). Stimuli entered the epifluorescence port of an Olympus BX51 upright microscope (Olympus–Life Science Solutions) and were projected onto the retinas through UV-compliant objective lenses. A 10x UPlanFLN/0.3 projected stimuli on to isolated larval eyes and a 4x UPlanSApo/0.16 projected stimuli onto adult eyecups. A background beam infrared (IR, RG780 filter) was projected onto the eyes through a second light path as the ‘neutral’ background for infrared visualization of eye and electrode placement using an infrared camera (QImaging, Retiga-2000RV) and Metamorph (Molecular Devices). The microscope was positioned over the chamber with a translation stage (Sutter Instrument, MT-800) and microelectrodes were inserted into eyes (or eyecups) with a micro-positioner (Sutter Instrument, MPC-385). Microelectrode signals were amplified by 10,000 (World Precision Instruments, DAM80, 0.1 Hz-1k Hz bandpass), and digitized (2000 Hz) with an Axon instruments 1440A (Molecular Devices) using Clampex 10 software. The 280 ERGs within a spectral dataset were saved as a single Clampex file by using the averaging option and retaining all the elements of the average.

### Spectral stimulus protocol

The 17-min spectral protocol was a fixed sequence of 280, 300 msec, monochromatic light flashes of different irradiances delivered through house software. The 64 unique stimuli and 6 replicates within the protocol created the spectral dataset, defined in Nelson 2019 [18] as a set of 70, 4X-averaged, ERG responses. Wavelengths were given in the order 650, 570, 490, 410, 330, 650, 610, 530, 450, 370 nm with 650 nm repeated as an index of response stability. At each wavelength 7 irradiances were delivered in 0.5 log unit increments, with brightness levels pre-adjusted to cover the anticipated response range from threshold to saturation. The interval between stimuli varied between 2.5 and 6 s, with the longer intervals separating the brighter stimuli. The protocol setting for maximal irradiances in log(quanta·μm^-2^·s^-1^) at each wavelength were 7.2 (650 nm), 6.3 (610 nm), 6.4 (570 nm), 6.3 (530 nm), 6.4 (490 nm), 6.1 (450 nm), 5.7 (410 nm), 5.7 (370 nm), and 5.2 (330 nm).

### Analysis of spectral data

Clampex files of spectral datasets were imported into Origin (various versions, Originlabs) for data analysis using scripts written in Origin Labtalk, where the 4X replicates of the stimulus were averaged, noise was filtered (33 point, 16.5 msec running average), trough to peak amplitudes of PIII responses were calculated, and responses were associated with the wavelength and irradiance of stimulation. Datasets with unstable responses over the recording period were not included.

The contributions of signals from different spectral types of cone to the PIII ERG response were determined using the method of Nelson et al [18]. Datasets from many eyes were normalized to the peak response within each dataset and then combined into large ‘genotype-level’ datasets for 5–7 dpf larvae or for adults. These genotype-level datasets included hundreds to thousands of amplitude-wavelength-irradiance points collected from many eyes. Genotype-level datasets were fit to a spectral model (Eq 1) which extracted three physiological properties for each of the 4 spectral types of zebrafish cone. This provides a method to determine whether signals of individual cone types were altered by mutation, and in what way. The cone properties extracted were *Vmax*, the maximal amplitude contribution of the cone type to the PIII ERG, *k*, the irradiance required to half-saturate that cone’s response amplitude at the peak absorbance wavelength, and *λmax*, the spectral sensitivity maximum of that cone. The spectral model appears in Eq 1.

$$V=\frac{Vrmax*I}{\left(I+\frac{kr}{A\left(\lambda rmax,\lambda \right)}\right)}+\frac{Vgmax*I}{\left(I+\frac{kg}{A\left(\lambda gmax,\lambda \right)}\right)}+\frac{Vbmax*I}{\left(I+\frac{kb}{A\left(\lambda bmax,\lambda \right)}\right)}+\frac{Vumax*I}{\left(I+\frac{ku}{A\left(\lambda umax,\lambda \right)}\right)}.$$ Eq 1

*V* is the cone PIII trough to peak signal amplitude, *I* is the quantal stimulus irradiance, and *λ* is the stimulus wavelength. The subscripts *r*, *g*, *b*, and *u* refer to red (*LWS* opsin expressing), green (*RH2* opsin expressing), blue (*SWS2* opsin expressing), and UV (*SWS1* opsin expressing) cones respectively. There are altogether 12 cone parameters that might be fit, however in many cases, for example datasets from mutants that lack signals from a cone type, the number that could be fit was less. This was evidenced by failure of the fitting algorithm (Levenburg Marquardt, as supplied by Originlabs) to converge on a solution within 100 iterations, or by the parameter encountering a boundary condition (Table 5). In this case a fixed value based on previous experience [35] was substituted (Table 5).

**Table 5:** Boundaries and seed values for cone fit parameters.

Fit parameter	Units	Fixed value	Lower bound	Upper bound
log *kr*	log(quanta·μm^-2^·s^-1^)	4.5	1	6
log *kg*	log(quanta·μm^-2^·s^-1^)	4.0	1	6
log *kb*	log(quanta·μm^-2^·s^-1^)	3.5	1	6
log *ku*	log(quanta·μm^-2^·s^-1^)	3.0	1	6

If Eq 1 curve fits lie outside these boundaries, a fixed value of semi-saturation for red (*kr*), green (*kg*), blue (*kb)*, or UV (*ku*) cones is substituted.

A(*λmax*, *λ*) is the Dartnall nomogram function [36] giving the spectral shape for zebrafish cone opsins. It is based in the observation that, on a reciprocal wavelength axis, the absorbance shapes of opsins with different absorbance maxima are closely similar. These nomogram shapes are expressed as order 8 polynomials. The polynomial of Hughes et al [37] is used for *r*, *g*, and *b* cones and the polynomial of Palacios et al [38] is used for *u* cones, which are slightly narrower in spectral shape. All nomograms are based in the cone spectral patch recordings of Palacios et al on *Giant Danio* [38], a species closely related to zebrafish. The polynomials are normalized, which has the effect of referring the fitted *k* (semisaturation irradiance) values for cones to *λmax*, the wavelength of maximal opsin absorbance. Nomogram coefficients are reproduced in Table 6.

**Table 6:** Polynomial coefficients for cone opsin absorbance nomograms.

Coefficient	r, g, b cones Hughes et al, 1998 (34)	UV cones Palacios et al, 1996 (35)
*C*_0_	−93.262	−48,139.554
*C*_1_	617.458	309,007.504
*C*_2_	−2,815.735	−770,875.542
*C*_3_	7,339.277	834,365.023
*C*_4_	−10,714.545	−57,657.482
*C*_5_	9,048.982	−815,689.388
*C*_6_	−4,410.667	871,189.984
*C*_7_	1,154.235	−389,189.665
*C*_8_	−125.738	66,989.122

Coefficents for order 8 polynomials representing normalized absorbance nomograms for red, green, and blue cones (r, g, b) and UV cones. These polynomial functions are represented as A(λ_max_, λ) in Eq 1.

### Confocal imaging

Larval zebrafish are moved from egg water with methylene blue into a petri dish with 20mL of 300μM PTU at 1-day post fertilization. Larvae are sorted into fluorescent and non-fluorescent phenotypes using the same methods as non-PTU larvae. At 5 days past fertilization larvae are placed into a separate petri dish and anesthetized with tricaine, as above. Low melting 0.8% agarose gel (Sigma-Aldrich, A0701) is melted on a hot plate, cooled to 35° C, and a drop is placed into a Lab-Tek II 2-chambered cover-glass bottom well for microscopy (ThermoFisher Scientific, 155279PK). Each of the two chambers is divided in 4 by an insert, giving a total of 8 chambers. A larva is dropped into the well followed by a second drop of agarose. The larval eye is pressed flat against the bottom of the well with forceps and the agarose is allowed to cool and harden. A drop of tricaine was added to the top to keep the larvae anesthetized during imaging. A ZEISS LSM T-PMT confocal microscope was used with Zen image software (version 2.3). The DPSS 561–10 (561 nm) laser was used to fluoresce the tdTomato fluorophore. The mYFP fluorescent protein was imaged with an Argon 488 laser. Z-stack image sets were collected at 1 μm intervals with a 25x objective and later analyzed using ImageJ (FIJI).

### Optomotor response

Larvae between the ages of 5 and 12 dpf were tested for optomotor response (OMR) in a 3.5-inch Petri dish placed directly on a laptop computer monitor (10–30 larvae per dish). The stimulus was generated using the PsychoPy Python library package through PyCharm (v. 2018). Colored gratings swept from left to right on the monitor with a spatial frequency of 0.36 cycles/cm, size of 50, band width of 20mm, and phase of 0.03 (50mm/sec). Two stimuli were given: black/white and red/black. The black/white stimulus was given first, to isolate responsive larvae. The red/black stimulus was next presented to test for red-blind mutants. The stimulus lasted for 60 seconds and larvae were given a minute break between stimuli in a dark incubator. While the wavelength emission peaks for monitor colors are not specifically known, in general red is ~610 nm; green, ~550 nm, and blue, ~450 nm [30]. The monitors do not stimulate zebrafish UV cones. Videos and images were taken with an iPhone 8 Plus camera. Before and after images were analyzed using Mac Preview.

### Optokinetic response

Larvae between the ages of 7 and 9 dpf were tested for optokinetic response (OKR). A larva was placed in a drop of 4% methyl cellulose [21] on a glass lantern slide on the computer monitor. Pinwheel stimuli were created using the PsychoPy Python package through PyCharm (v. 2018). The pinwheel stimulus was made up of 12 alternating color wedges with the larvae at the center of the rotating stimulus. The colors included: black/white, red/blue, red/black, and green/black. The pinwheel rotated at 7.5 revolutions per minute. Correct eye movements were counted as a sweeping movement clockwise and snapping back counterclockwise. The numbers of correct eye movements were counted over a 60 second period.

### Immunohistochemistry of Larvae

Larvae fixed in 4% paraformaldehyde / 0.1M phosphate buffered saline (Electron Microscopy Sciences, catalogue 15710; Omnipur, catalogue 6505; PFA/PBS) for 30 min were dissected in a Petri dish filled with PBS using two 30-G disposable syringe needles to isolate retinas. The retinas were then transferred into 1.5 mL Eppendorf tubes (ThermoScientific, catalogue 3451) for subsequent blocking with lamb serum blocking buffer (Life Technolgies/Invitrogen, catalogue 6640), washing with Tween wash buffer (Invitrogen, catalogue 00–3005), and incubated with 1D4 anti zebrafish red-cone opsin primary antibody (AbCam, catalog ab5417, 1:100) [39] and Alexa Fluor488 anti-mouse secondary antibody (Life Technologies/Invitrogen, catalogue 16070096, 1:100). The primary antibody was left for an incubation period of 3–4 days while the secondary was left for 1–2 days. Retinas were then mounted with Vectashield Antifade Mounting Medium with DAPI (Vector Laboratories, catalogue H-1200) between two coverslips separated by a spacer-square of black duct tape with a paper-punch hole in the middle to accommodate the retina. The cover glass sandwich was pasted to a microscope slide using clear nail polish. The retinas were imaged on the same confocal microscope used for live imaging with the Argon (458, 488, 514), Diode (405–30) and DPSS 561–10 lasers. Images were analyzed with ImageJ (FIJI).

Adult eye cups were dissected and fixed in PFA/PBS for one hour and washed with PBS. Retinas were removed from the eye cups using No. 5 forceps to peel away the outside layers of the eye cup. Retinas were then placed in a 1.5 mL Eppendorfs for the same process of staining as with the larvae. Zpr-1 (Zebrafish International Resource Center, ZFIN ID: ZDB-ATB-081002-43, 1:100) was used in the first round of staining with Alexa Fluor594 anti-mouse (Thermo Fisher Scientific, catalog A-21135, 1:100) secondary following the same timeline as previously mentioned. The second round of staining included 1D4 anti-red-opsin primary and Alexa Fluor488 anti-mouse secondary. Retinas were mounted on a coverglass fixed into place with a drop of 0. 8% agarose as above and mounted with VECTASHIELD Vibrance Antifade Mounting Medium. The samples were imaged and analyzed using the same methods as the larvae.

### Quantitative RT-PCR of RH2 opsins in trβ2 6BP+1 mutants, heterozygotes, and wild types

Total RNA was isolated from zebrafish retinal tissues using RNeasy Plus Mini Kit (Qiagen), the retinal tissues were collected from wild type, heterozygous mutant and homozygous mutant adult zebrafish. Reverse transcription was carried out with SuperScript III First-Strand Synthesis system (Invitrogen, Grand Island, NY) using 1μg of RNA. Quantitative real-time RT-PCR was performed on a ViiA 7 Real-Time PCR System (Applied Biosystems Inc.) The PCR was performed as follows: 2 min 30 sec at 95⁰ C for enzyme activation followed by 40 cycles of 15 s at 95⁰ C, and 1 min at 60⁰ C. Melting curve analysis was performed to confirm the real-time PCR products. All quantifications were normalized to 18S rRNA levels. Primer sequences used are: RH2-1/RH2-2FW: ttaacaggggagctgctttc. RH2-1/RH2-2RV: cttgccacagaaaagggtgt.

RH2-4FW: caaattttctgccagtcacg. RH2-4RV: ctggtccacatgaacactgc.

GAPDH-FW: aagaaacagcaaaggggtca. GAPDH-RV: tgctggtattgctctcaacg.

### Randomization/Blind, Inclusion/Exclusion, sample size estimation

Heterozygous in-cross or heterozygous mutant spawns resulted in 2–3 genotypes including mutants, heterozygotes, and WTs. The genotype was unknown when the fish were selected for physiological experimentation. Following physiological data collection, tail snip samples were given letter number names and sequenced without knowledge of physiological results. Optokinetic experiments were conducted without knowing the genotype of the larvae, but genotypes were known for the optomotor experiments. Live confocal imaging was conducted without known genotypes as we could not discern WTs from heterozygotes in the fluorescent group or any of the non-fluorescing fish. Genotypes of larval zebrafish were unknown before antibody staining, while adult zebrafish genotypes were known before staining.

We excluded physiological datasets with unstable response amplitudes over the course of the 17-minute protocol. All datasets that maintained stable response amplitudes were included. Unstable datasets could include either loss of electrode penetration or gradual declines/increases in amplitude. We excluded parameter fits that did not converge in the model and typical fixed values were substituted.

Previous studies have shown that 10 eyes/1000 amplitudes (spectral responses/data points) are enough to make the physiological distinctions expected [18].
